# Supplementary material for: The targetable kinase PIM1 drives ALK inhibitor resistance in high-risk neuroblastoma independent of MYCN status
Source: Nat Commun. 2019 Nov 28;10:5428. doi: 10.1038/s41467-019-13315-x (PMC6883072; doi:10.1038/s41467-019-13315-x)
Supplement: Supplementary file 1 — Supplementary Information [file 41467_2019_13315_MOESM1_ESM.pptx]

## Slide 1
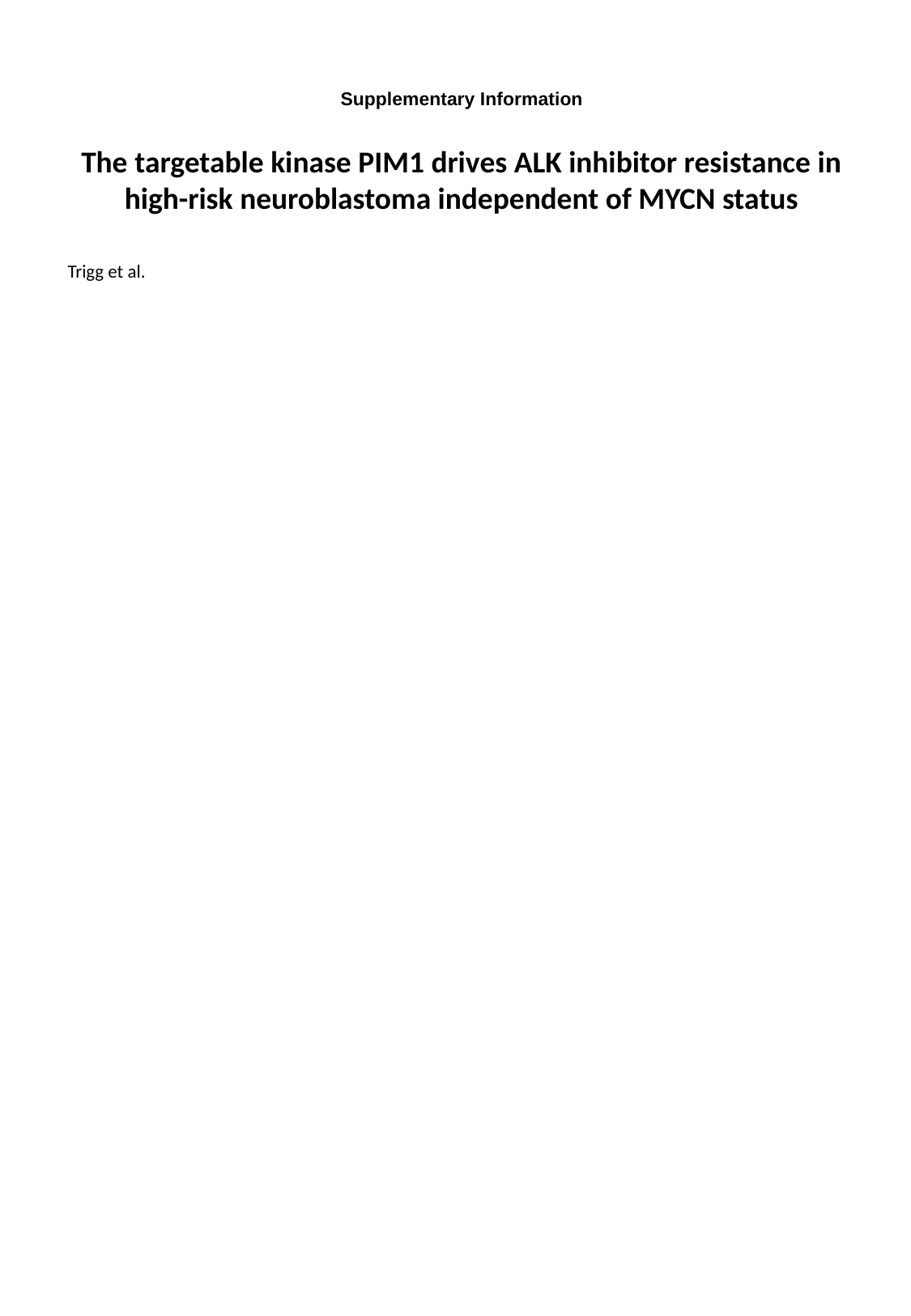

Supplementary Information
The targetable kinase PIM1 drives ALK inhibitor resistance in high-risk neuroblastoma independent of MYCN status
Trigg et al.

## Slide 2
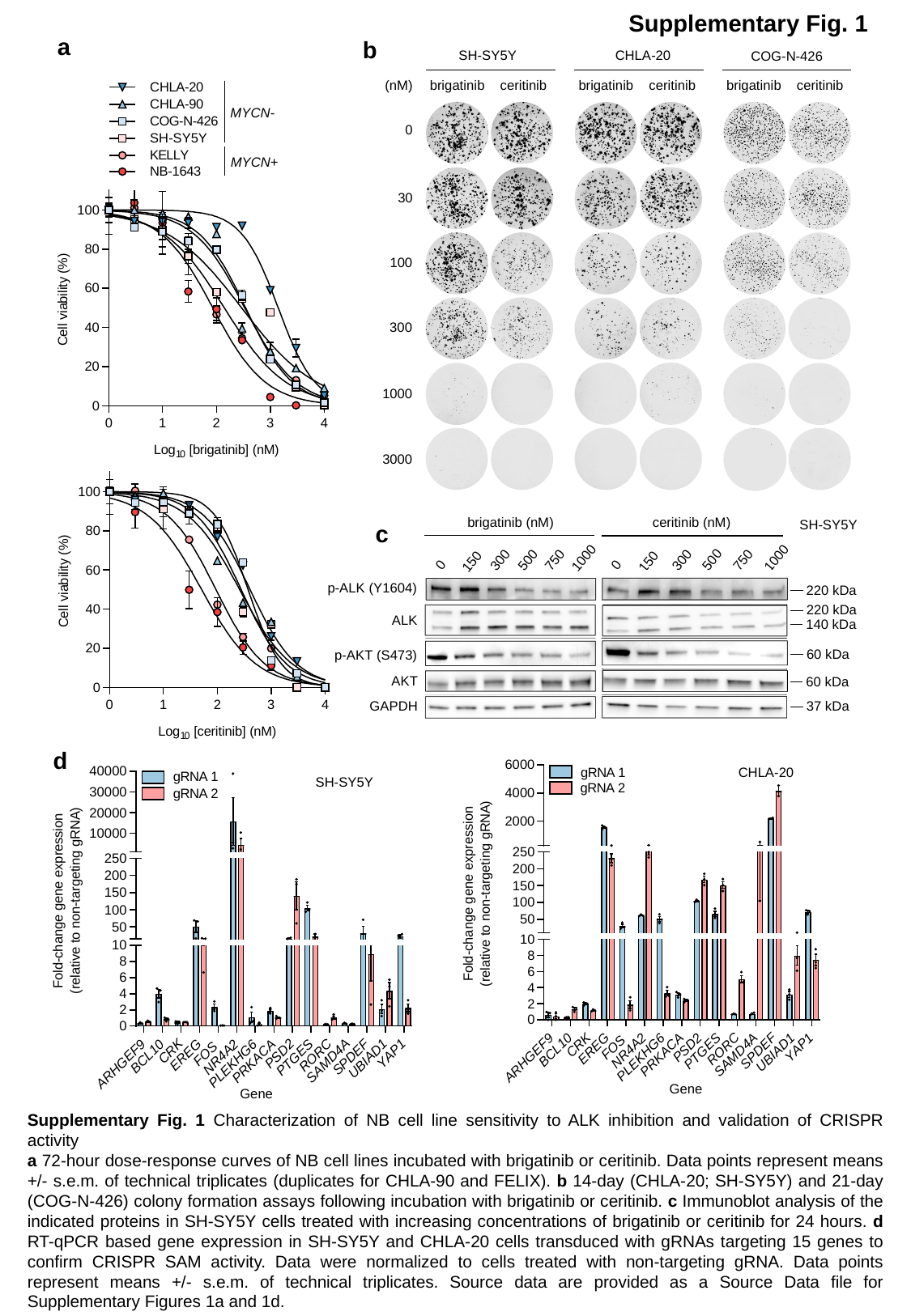

Supplementary Fig. 1
a
b
CHLA-20
brigatinib
ceritinib
SH-SY5Y
brigatinib
ceritinib
COG-N-426
brigatinib
ceritinib
(nM)
0
30
100
300
1000
3000
MYCN-
MYCN+
ceritinib (nM)
brigatinib (nM)
SH-SY5Y
c
1000
1000
750
500
750
500
300
300
150
150
0
0
p-ALK (Y1604)
220 kDa
220 kDa
ALK
140 kDa
60 kDa
60 kDa
37 kDa
p-AKT (S473)
AKT
GAPDH
d
CHLA-20
SH-SY5Y
Gene
Gene
Supplementary Fig. 1 Characterization of NB cell line sensitivity to ALK inhibition and validation of CRISPR activity
a 72-hour dose-response curves of NB cell lines incubated with brigatinib or ceritinib. Data points represent means +/- s.e.m. of technical triplicates (duplicates for CHLA-90 and FELIX). b 14-day (CHLA-20; SH-SY5Y) and 21-day (COG-N-426) colony formation assays following incubation with brigatinib or ceritinib. c Immunoblot analysis of the indicated proteins in SH-SY5Y cells treated with increasing concentrations of brigatinib or ceritinib for 24 hours. d RT-qPCR based gene expression in SH-SY5Y and CHLA-20 cells transduced with gRNAs targeting 15 genes to confirm CRISPR SAM activity. Data were normalized to cells treated with non-targeting gRNA. Data points represent means +/- s.e.m. of technical triplicates. Source data are provided as a Source Data file for Supplementary Figures 1a and 1d.

## Slide 3
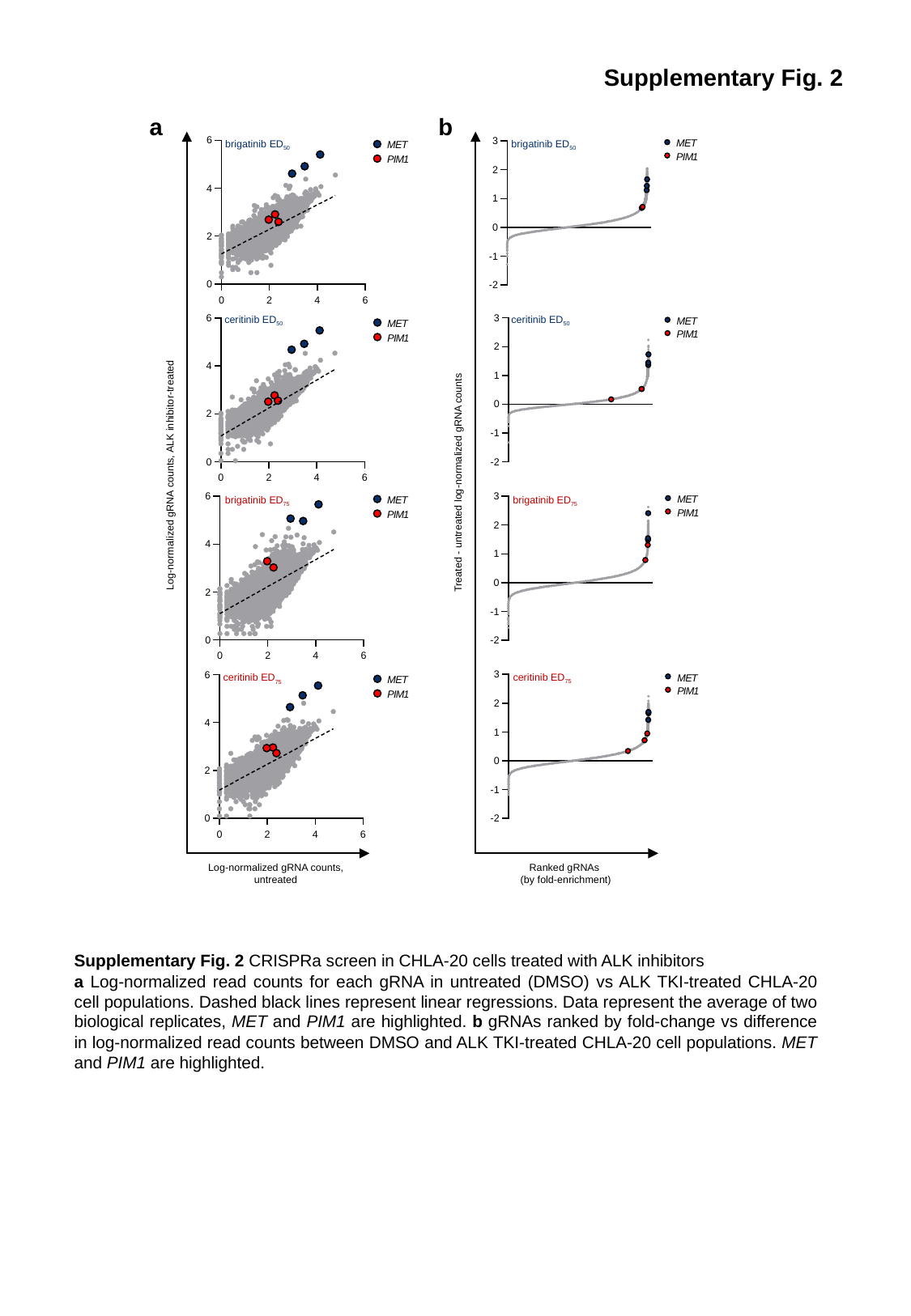

Supplementary Fig. 2
b
brigatinib ED50
ceritinib ED50
brigatinib ED75
ceritinib ED75
Treated - untreated log-normalized gRNA counts
Ranked gRNAs
(by fold-enrichment)
a
brigatinib ED50
ceritinib ED50
Log-normalized gRNA counts, ALK inhibitor-treated
brigatinib ED75
ceritinib ED75
Log-normalized gRNA counts, untreated
Supplementary Fig. 2 CRISPRa screen in CHLA-20 cells treated with ALK inhibitors
a Log-normalized read counts for each gRNA in untreated (DMSO) vs ALK TKI-treated CHLA-20 cell populations. Dashed black lines represent linear regressions. Data represent the average of two biological replicates, MET and PIM1 are highlighted. b gRNAs ranked by fold-change vs difference in log-normalized read counts between DMSO and ALK TKI-treated CHLA-20 cell populations. MET and PIM1 are highlighted.

## Slide 4
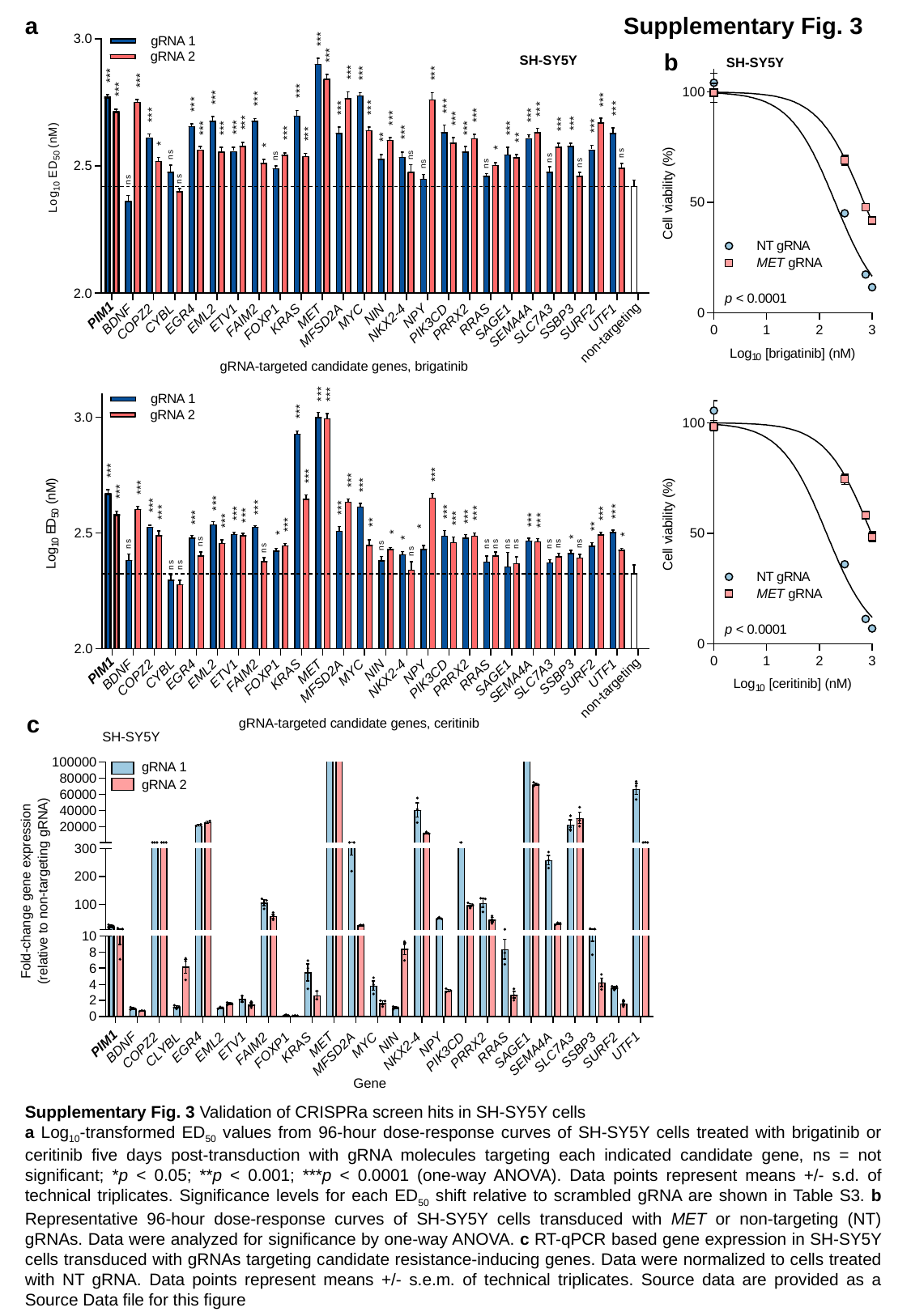

a
b
c
SH-SY5Y
SH-SY5Y
SH-SY5Y
Supplementary Fig. 3
Gene
Supplementary Fig. 3 Validation of CRISPRa screen hits in SH-SY5Y cells
a Log10-transformed ED50 values from 96-hour dose-response curves of SH-SY5Y cells treated with brigatinib or ceritinib five days post-transduction with gRNA molecules targeting each indicated candidate gene, ns = not significant; *p < 0.05; **p < 0.001; ***p < 0.0001 (one-way ANOVA). Data points represent means +/- s.d. of technical triplicates. Significance levels for each ED50 shift relative to scrambled gRNA are shown in Table S3. b Representative 96-hour dose-response curves of SH-SY5Y cells transduced with MET or non-targeting (NT) gRNAs. Data were analyzed for significance by one-way ANOVA. c RT-qPCR based gene expression in SH-SY5Y cells transduced with gRNAs targeting candidate resistance-inducing genes. Data were normalized to cells treated with NT gRNA. Data points represent means +/- s.e.m. of technical triplicates. Source data are provided as a Source Data file for this figure

## Slide 5
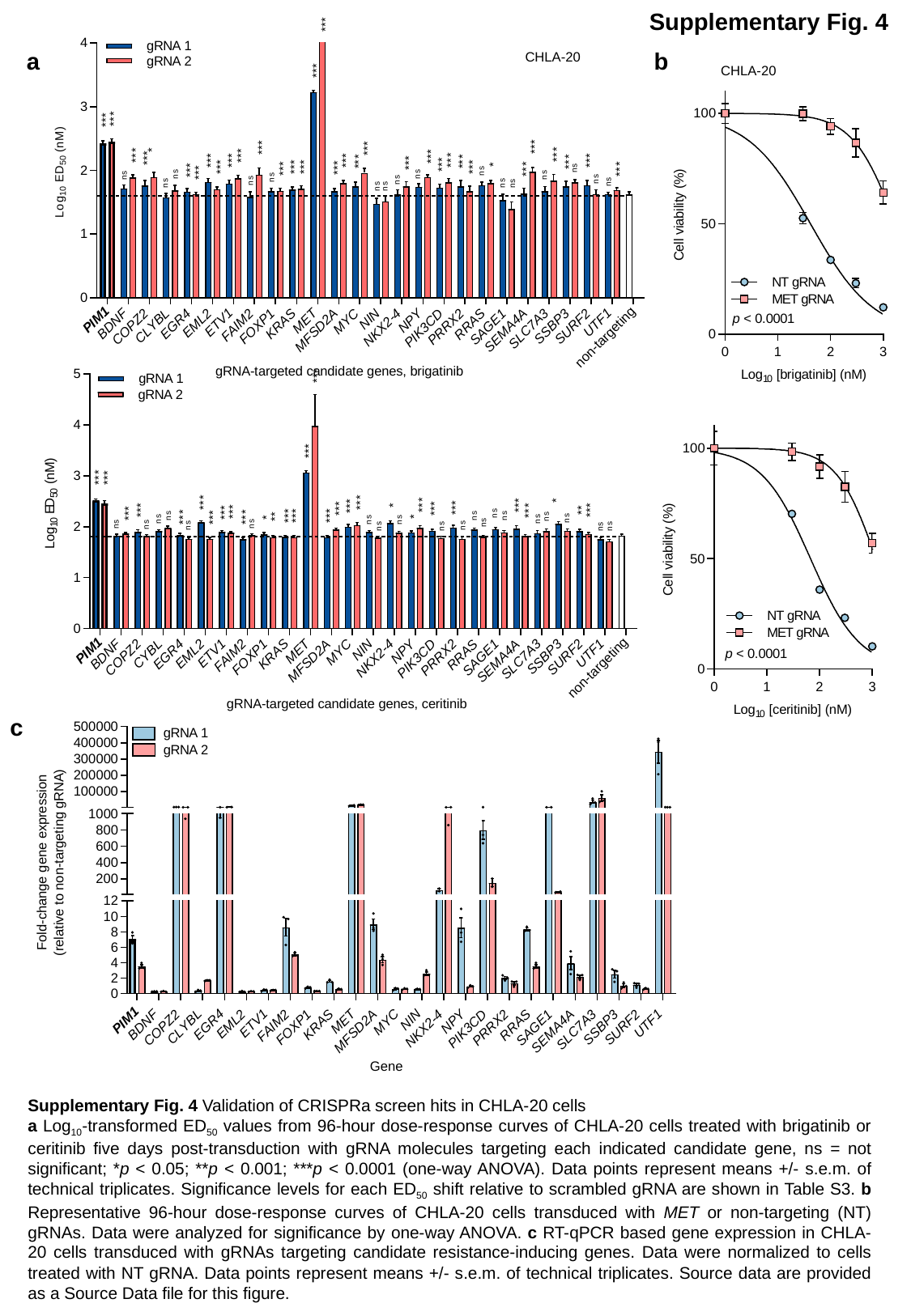

Supplementary Fig. 4
a
b
CHLA-20
CHLA-20
c
Gene
Supplementary Fig. 4 Validation of CRISPRa screen hits in CHLA-20 cells
a Log10-transformed ED50 values from 96-hour dose-response curves of CHLA-20 cells treated with brigatinib or ceritinib five days post-transduction with gRNA molecules targeting each indicated candidate gene, ns = not significant; *p < 0.05; **p < 0.001; ***p < 0.0001 (one-way ANOVA). Data points represent means +/- s.e.m. of technical triplicates. Significance levels for each ED50 shift relative to scrambled gRNA are shown in Table S3. b Representative 96-hour dose-response curves of CHLA-20 cells transduced with MET or non-targeting (NT) gRNAs. Data were analyzed for significance by one-way ANOVA. c RT-qPCR based gene expression in CHLA-20 cells transduced with gRNAs targeting candidate resistance-inducing genes. Data were normalized to cells treated with NT gRNA. Data points represent means +/- s.e.m. of technical triplicates. Source data are provided as a Source Data file for this figure.

## Slide 6
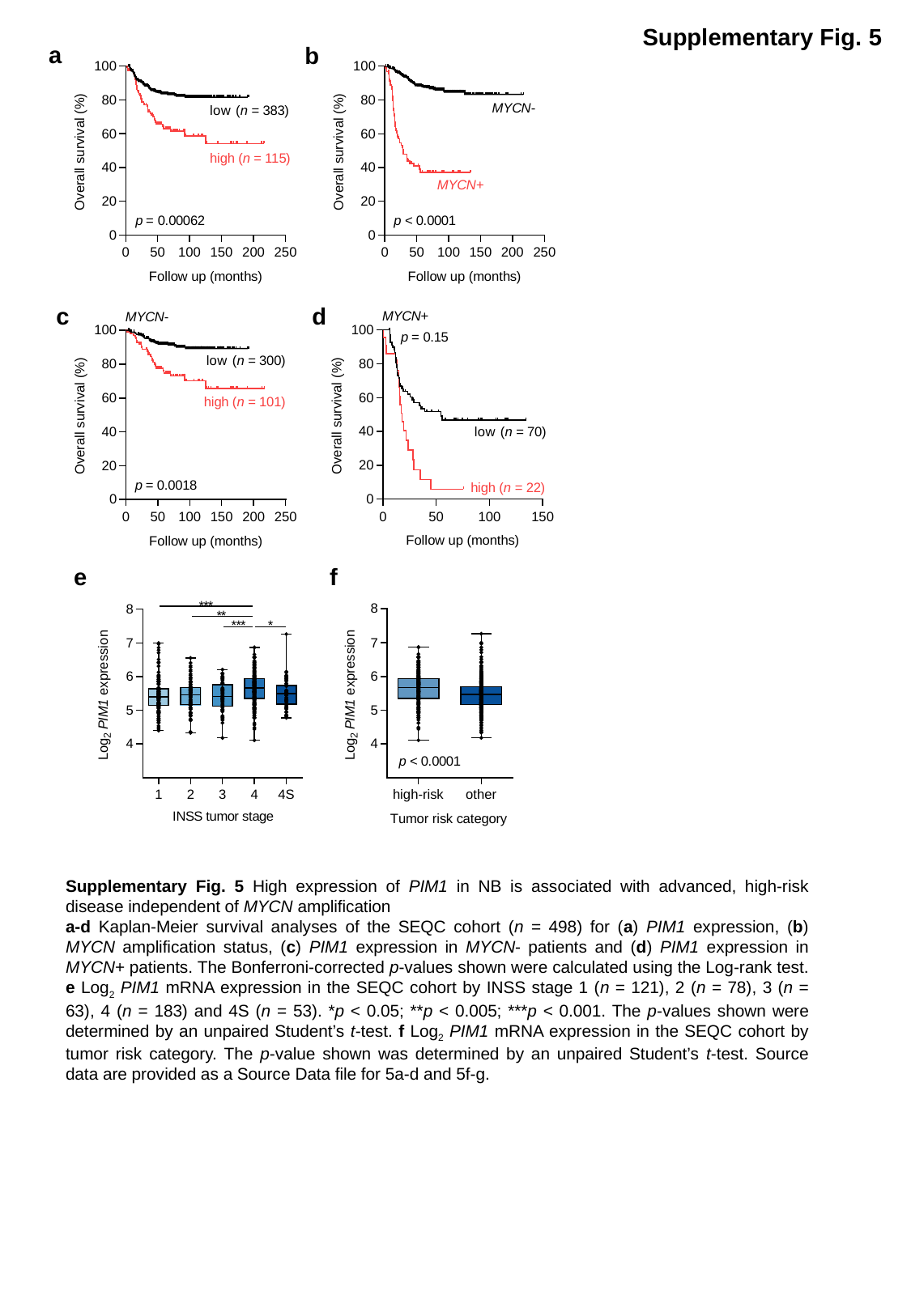

Supplementary Fig. 5
a
b
c
d
f
e
Supplementary Fig. 5 High expression of PIM1 in NB is associated with advanced, high-risk disease independent of MYCN amplification
a-d Kaplan-Meier survival analyses of the SEQC cohort (n = 498) for (a) PIM1 expression, (b) MYCN amplification status, (c) PIM1 expression in MYCN- patients and (d) PIM1 expression in MYCN+ patients. The Bonferroni-corrected p-values shown were calculated using the Log-rank test. e Log2 PIM1 mRNA expression in the SEQC cohort by INSS stage 1 (n = 121), 2 (n = 78), 3 (n = 63), 4 (n = 183) and 4S (n = 53). *p < 0.05; **p < 0.005; ***p < 0.001. The p-values shown were determined by an unpaired Student’s t-test. f Log2 PIM1 mRNA expression in the SEQC cohort by tumor risk category. The p-value shown was determined by an unpaired Student’s t-test. Source data are provided as a Source Data file for 5a-d and 5f-g.

## Slide 7
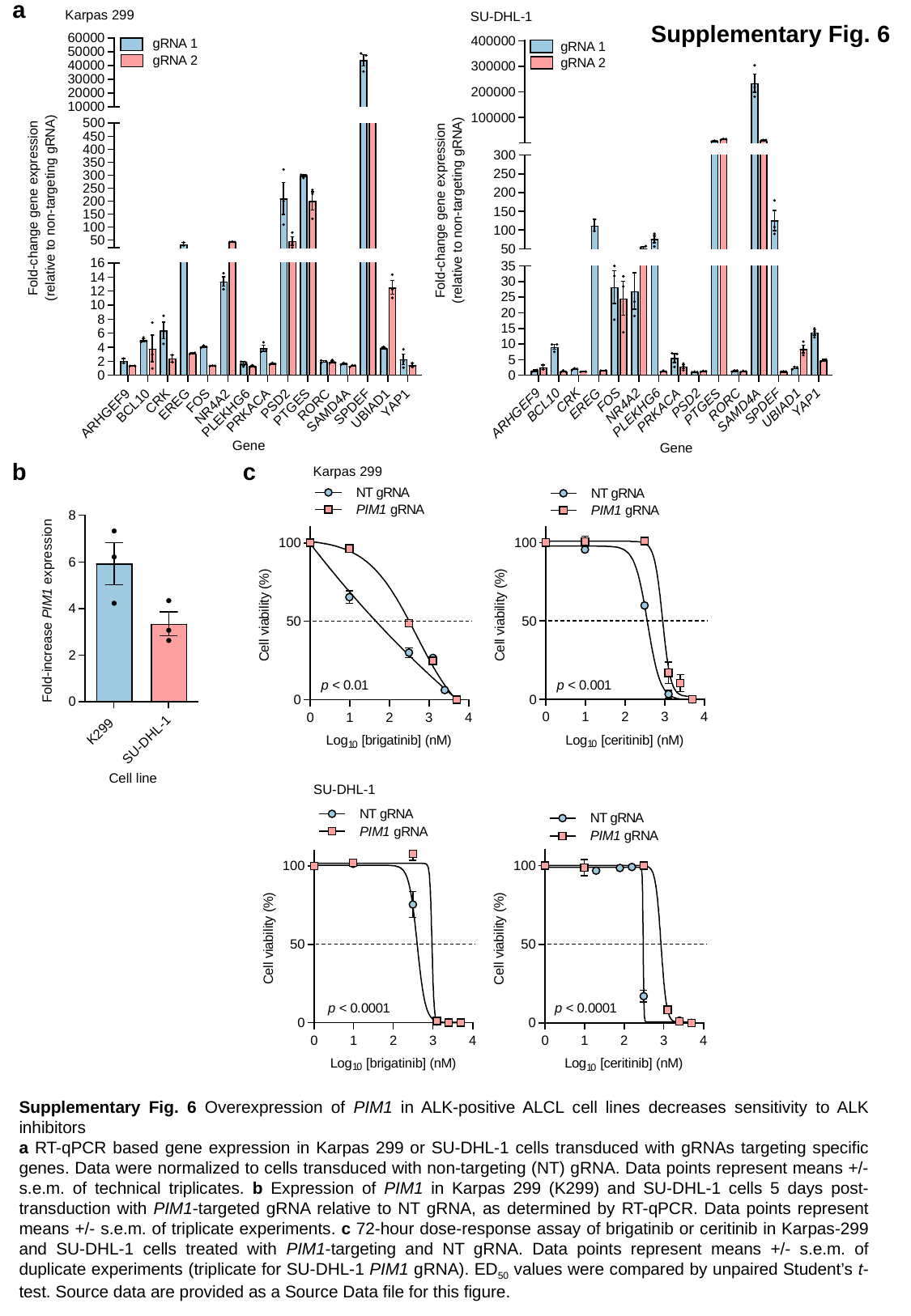

a
Karpas 299
SU-DHL-1
c
Karpas 299
SU-DHL-1
b
Supplementary Fig. 6
Gene
Gene
Cell line
Supplementary Fig. 6 Overexpression of PIM1 in ALK-positive ALCL cell lines decreases sensitivity to ALK inhibitors
a RT-qPCR based gene expression in Karpas 299 or SU-DHL-1 cells transduced with gRNAs targeting specific genes. Data were normalized to cells transduced with non-targeting (NT) gRNA. Data points represent means +/- s.e.m. of technical triplicates. b Expression of PIM1 in Karpas 299 (K299) and SU-DHL-1 cells 5 days post-transduction with PIM1-targeted gRNA relative to NT gRNA, as determined by RT-qPCR. Data points represent means +/- s.e.m. of triplicate experiments. c 72-hour dose-response assay of brigatinib or ceritinib in Karpas-299 and SU-DHL-1 cells treated with PIM1-targeting and NT gRNA. Data points represent means +/- s.e.m. of duplicate experiments (triplicate for SU-DHL-1 PIM1 gRNA). ED50 values were compared by unpaired Student’s t-test. Source data are provided as a Source Data file for this figure.

## Slide 8
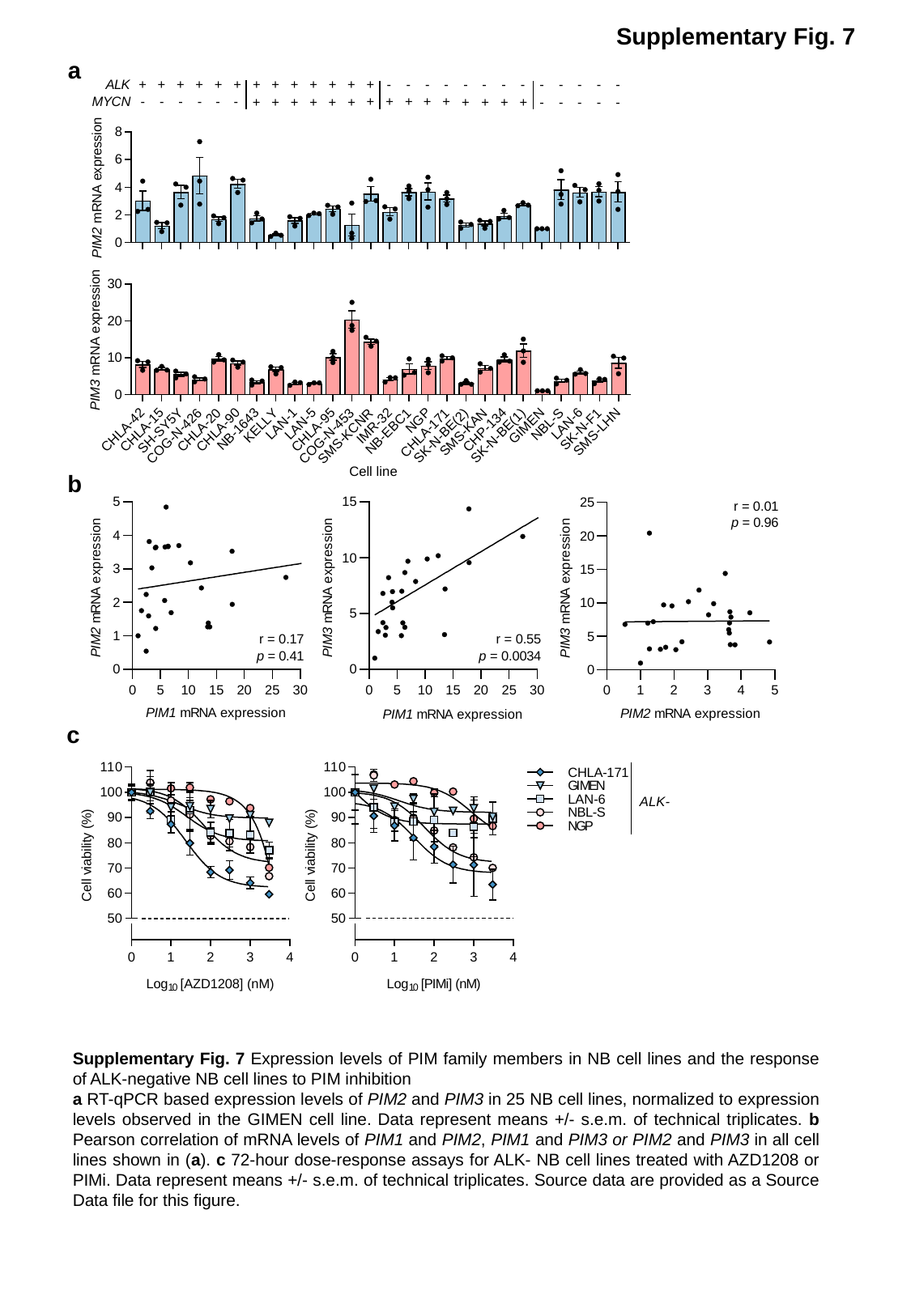

Supplementary Fig. 7
a
b
c
ALK-
Cell line
Supplementary Fig. 7 Expression levels of PIM family members in NB cell lines and the response of ALK-negative NB cell lines to PIM inhibition
a RT-qPCR based expression levels of PIM2 and PIM3 in 25 NB cell lines, normalized to expression levels observed in the GIMEN cell line. Data represent means +/- s.e.m. of technical triplicates. b Pearson correlation of mRNA levels of PIM1 and PIM2, PIM1 and PIM3 or PIM2 and PIM3 in all cell lines shown in (a). c 72-hour dose-response assays for ALK- NB cell lines treated with AZD1208 or PIMi. Data represent means +/- s.e.m. of technical triplicates. Source data are provided as a Source Data file for this figure.

## Slide 9
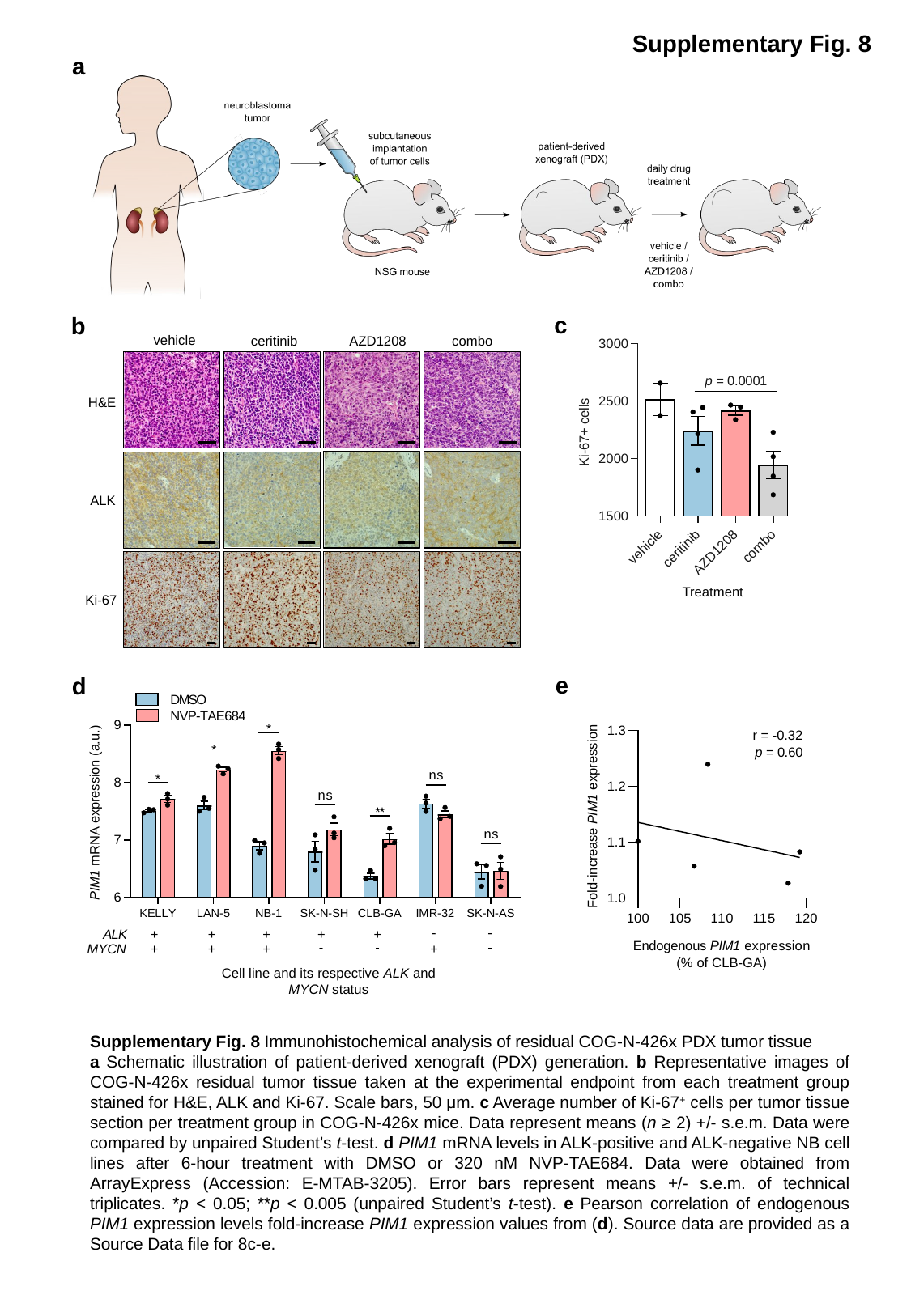

Supplementary Fig. 8
a
b
Ki-67
H&E
ALK
vehicle
ceritinib
AZD1208
combo
c
e
d
Treatment
Cell line and its respective ALK and MYCN status
Supplementary Fig. 8 Immunohistochemical analysis of residual COG-N-426x PDX tumor tissue
a Schematic illustration of patient-derived xenograft (PDX) generation. b Representative images of COG-N-426x residual tumor tissue taken at the experimental endpoint from each treatment group stained for H&E, ALK and Ki-67. Scale bars, 50 μm. c Average number of Ki-67+ cells per tumor tissue section per treatment group in COG-N-426x mice. Data represent means (n ≥ 2) +/- s.e.m. Data were compared by unpaired Student’s t-test. d PIM1 mRNA levels in ALK-positive and ALK-negative NB cell lines after 6-hour treatment with DMSO or 320 nM NVP-TAE684. Data were obtained from ArrayExpress (Accession: E-MTAB-3205). Error bars represent means +/- s.e.m. of technical triplicates. *p < 0.05; **p < 0.005 (unpaired Student’s t-test). e Pearson correlation of endogenous PIM1 expression levels fold-increase PIM1 expression values from (d). Source data are provided as a Source Data file for 8c-e.

## Slide 10
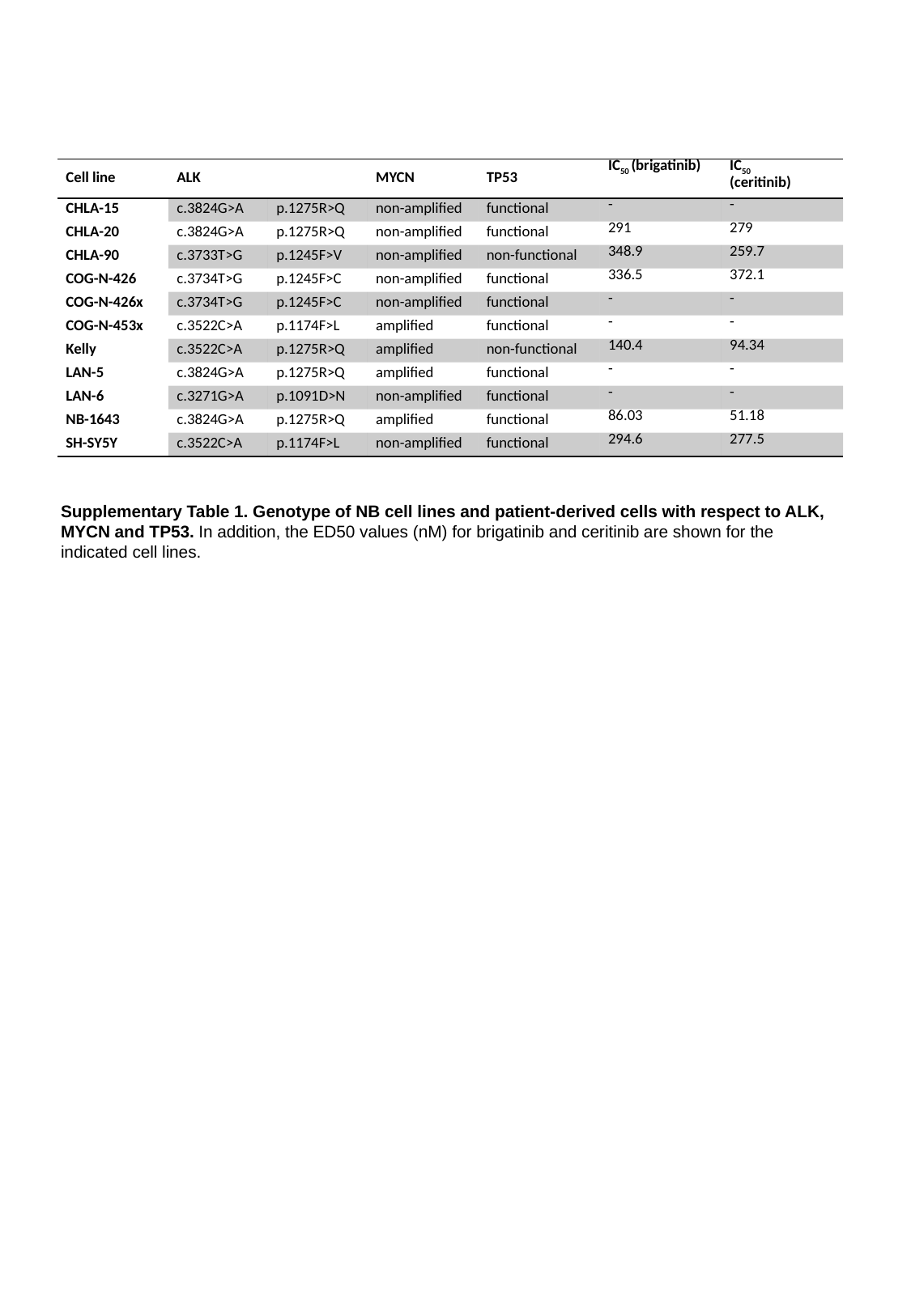

| Cell line | ALK | | MYCN | TP53 | IC50 (brigatinib) | IC50 (ceritinib) |
| --- | --- | --- | --- | --- | --- | --- |
| CHLA-15 | c.3824G>A | p.1275R>Q | non-amplified | functional | - | - |
| CHLA-20 | c.3824G>A | p.1275R>Q | non-amplified | functional | 291 | 279 |
| CHLA-90 | c.3733T>G | p.1245F>V | non-amplified | non-functional | 348.9 | 259.7 |
| COG-N-426 | c.3734T>G | p.1245F>C | non-amplified | functional | 336.5 | 372.1 |
| COG-N-426x | c.3734T>G | p.1245F>C | non-amplified | functional | - | - |
| COG-N-453x | c.3522C>A | p.1174F>L | amplified | functional | - | - |
| Kelly | c.3522C>A | p.1275R>Q | amplified | non-functional | 140.4 | 94.34 |
| LAN-5 | c.3824G>A | p.1275R>Q | amplified | functional | - | - |
| LAN-6 | c.3271G>A | p.1091D>N | non-amplified | functional | - | - |
| NB-1643 | c.3824G>A | p.1275R>Q | amplified | functional | 86.03 | 51.18 |
| SH-SY5Y | c.3522C>A | p.1174F>L | non-amplified | functional | 294.6 | 277.5 |
Supplementary Table 1. Genotype of NB cell lines and patient-derived cells with respect to ALK, MYCN and TP53. In addition, the ED50 values (nM) for brigatinib and ceritinib are shown for the indicated cell lines.

## Slide 11
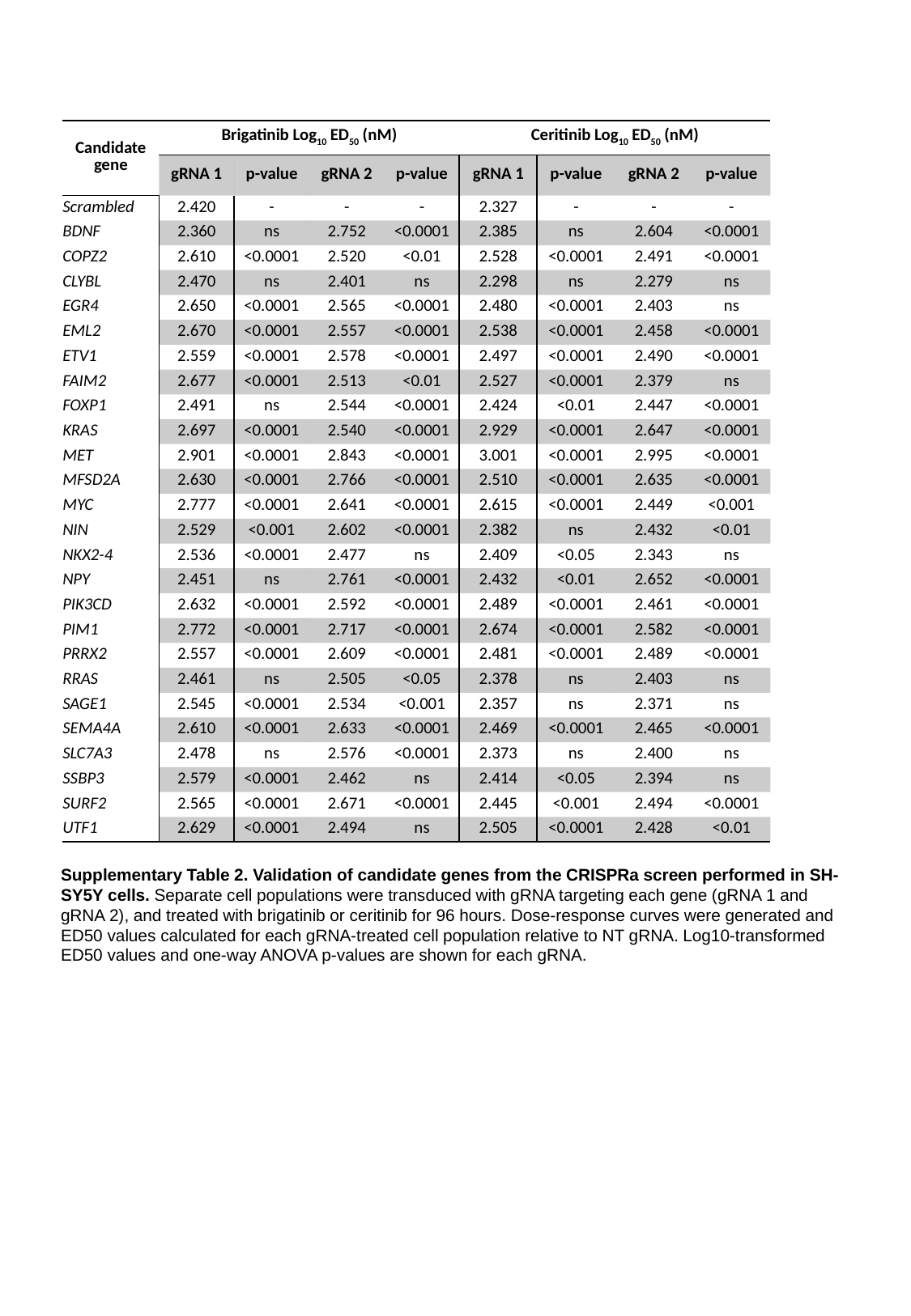

| Candidate gene | Brigatinib Log10 ED50 (nM) | | | | Ceritinib Log10 ED50 (nM) | | | |
| --- | --- | --- | --- | --- | --- | --- | --- | --- |
| | gRNA 1 | p-value | gRNA 2 | p-value | gRNA 1 | p-value | gRNA 2 | p-value |
| Scrambled | 2.420 | - | - | - | 2.327 | - | - | - |
| BDNF | 2.360 | ns | 2.752 | <0.0001 | 2.385 | ns | 2.604 | <0.0001 |
| COPZ2 | 2.610 | <0.0001 | 2.520 | <0.01 | 2.528 | <0.0001 | 2.491 | <0.0001 |
| CLYBL | 2.470 | ns | 2.401 | ns | 2.298 | ns | 2.279 | ns |
| EGR4 | 2.650 | <0.0001 | 2.565 | <0.0001 | 2.480 | <0.0001 | 2.403 | ns |
| EML2 | 2.670 | <0.0001 | 2.557 | <0.0001 | 2.538 | <0.0001 | 2.458 | <0.0001 |
| ETV1 | 2.559 | <0.0001 | 2.578 | <0.0001 | 2.497 | <0.0001 | 2.490 | <0.0001 |
| FAIM2 | 2.677 | <0.0001 | 2.513 | <0.01 | 2.527 | <0.0001 | 2.379 | ns |
| FOXP1 | 2.491 | ns | 2.544 | <0.0001 | 2.424 | <0.01 | 2.447 | <0.0001 |
| KRAS | 2.697 | <0.0001 | 2.540 | <0.0001 | 2.929 | <0.0001 | 2.647 | <0.0001 |
| MET | 2.901 | <0.0001 | 2.843 | <0.0001 | 3.001 | <0.0001 | 2.995 | <0.0001 |
| MFSD2A | 2.630 | <0.0001 | 2.766 | <0.0001 | 2.510 | <0.0001 | 2.635 | <0.0001 |
| MYC | 2.777 | <0.0001 | 2.641 | <0.0001 | 2.615 | <0.0001 | 2.449 | <0.001 |
| NIN | 2.529 | <0.001 | 2.602 | <0.0001 | 2.382 | ns | 2.432 | <0.01 |
| NKX2-4 | 2.536 | <0.0001 | 2.477 | ns | 2.409 | <0.05 | 2.343 | ns |
| NPY | 2.451 | ns | 2.761 | <0.0001 | 2.432 | <0.01 | 2.652 | <0.0001 |
| PIK3CD | 2.632 | <0.0001 | 2.592 | <0.0001 | 2.489 | <0.0001 | 2.461 | <0.0001 |
| PIM1 | 2.772 | <0.0001 | 2.717 | <0.0001 | 2.674 | <0.0001 | 2.582 | <0.0001 |
| PRRX2 | 2.557 | <0.0001 | 2.609 | <0.0001 | 2.481 | <0.0001 | 2.489 | <0.0001 |
| RRAS | 2.461 | ns | 2.505 | <0.05 | 2.378 | ns | 2.403 | ns |
| SAGE1 | 2.545 | <0.0001 | 2.534 | <0.001 | 2.357 | ns | 2.371 | ns |
| SEMA4A | 2.610 | <0.0001 | 2.633 | <0.0001 | 2.469 | <0.0001 | 2.465 | <0.0001 |
| SLC7A3 | 2.478 | ns | 2.576 | <0.0001 | 2.373 | ns | 2.400 | ns |
| SSBP3 | 2.579 | <0.0001 | 2.462 | ns | 2.414 | <0.05 | 2.394 | ns |
| SURF2 | 2.565 | <0.0001 | 2.671 | <0.0001 | 2.445 | <0.001 | 2.494 | <0.0001 |
| UTF1 | 2.629 | <0.0001 | 2.494 | ns | 2.505 | <0.0001 | 2.428 | <0.01 |
Supplementary Table 2. Validation of candidate genes from the CRISPRa screen performed in SH-SY5Y cells. Separate cell populations were transduced with gRNA targeting each gene (gRNA 1 and gRNA 2), and treated with brigatinib or ceritinib for 96 hours. Dose-response curves were generated and ED50 values calculated for each gRNA-treated cell population relative to NT gRNA. Log10-transformed ED50 values and one-way ANOVA p-values are shown for each gRNA.

## Slide 12
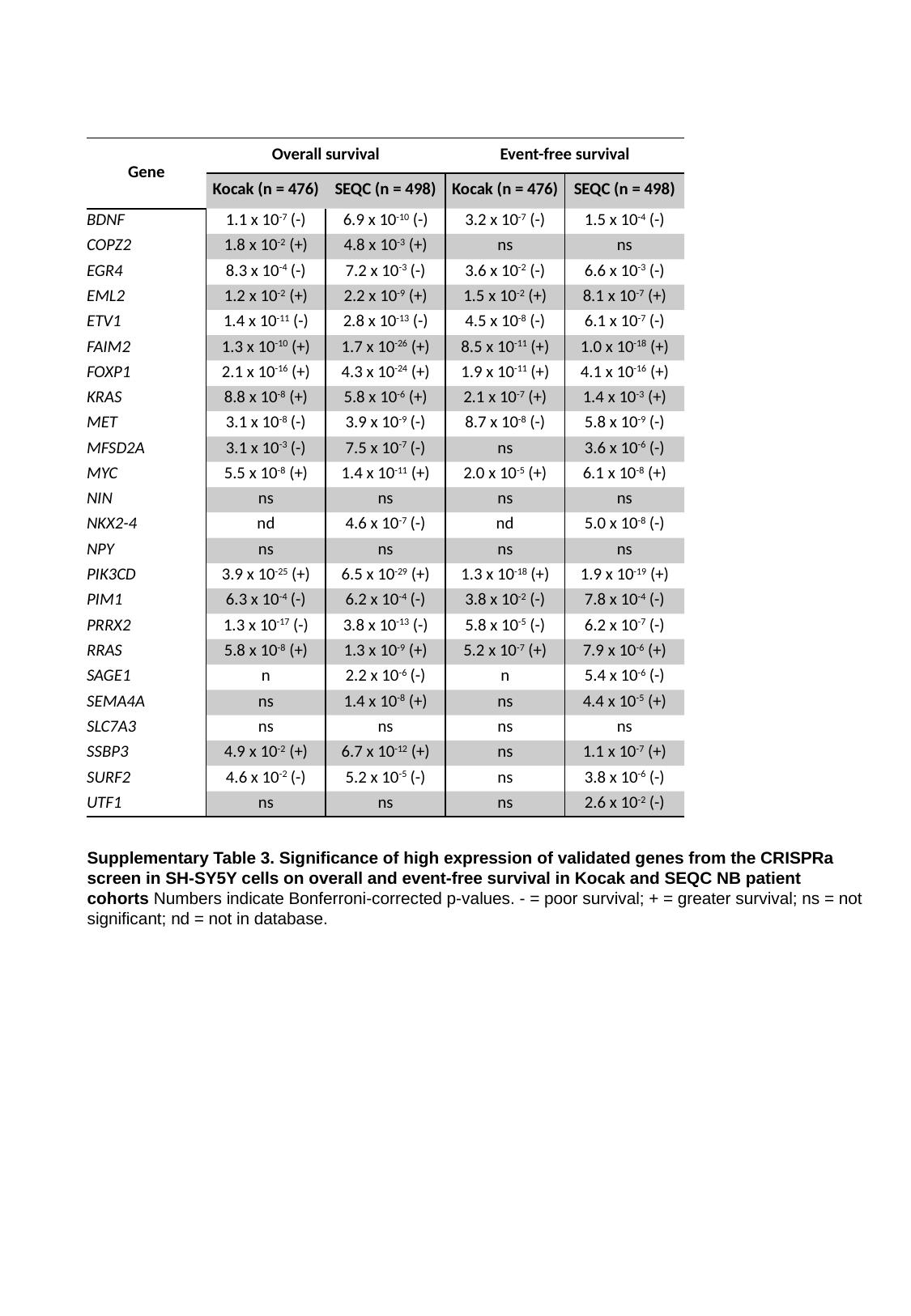

| Gene | Overall survival | | Event-free survival | |
| --- | --- | --- | --- | --- |
| | Kocak (n = 476) | SEQC (n = 498) | Kocak (n = 476) | SEQC (n = 498) |
| BDNF | 1.1 x 10^-7^ (-) | 6.9 x 10^-10^ (-) | 3.2 x 10^-7^ (-) | 1.5 x 10^-4^ (-) |
| COPZ2 | 1.8 x 10^-2^ (+) | 4.8 x 10^-3^ (+) | ns | ns |
| EGR4 | 8.3 x 10^-4^ (-) | 7.2 x 10^-3^ (-) | 3.6 x 10^-2^ (-) | 6.6 x 10^-3^ (-) |
| EML2 | 1.2 x 10^-2^ (+) | 2.2 x 10^-9^ (+) | 1.5 x 10^-2^ (+) | 8.1 x 10^-7^ (+) |
| ETV1 | 1.4 x 10^-11^ (-) | 2.8 x 10^-13^ (-) | 4.5 x 10^-8^ (-) | 6.1 x 10^-7^ (-) |
| FAIM2 | 1.3 x 10^-10^ (+) | 1.7 x 10^-26^ (+) | 8.5 x 10^-11^ (+) | 1.0 x 10^-18^ (+) |
| FOXP1 | 2.1 x 10^-16^ (+) | 4.3 x 10^-24^ (+) | 1.9 x 10^-11^ (+) | 4.1 x 10^-16^ (+) |
| KRAS | 8.8 x 10^-8^ (+) | 5.8 x 10^-6^ (+) | 2.1 x 10^-7^ (+) | 1.4 x 10^-3^ (+) |
| MET | 3.1 x 10^-8^ (-) | 3.9 x 10^-9^ (-) | 8.7 x 10^-8^ (-) | 5.8 x 10^-9^ (-) |
| MFSD2A | 3.1 x 10^-3^ (-) | 7.5 x 10^-7^ (-) | ns | 3.6 x 10^-6^ (-) |
| MYC | 5.5 x 10^-8^ (+) | 1.4 x 10^-11^ (+) | 2.0 x 10^-5^ (+) | 6.1 x 10^-8^ (+) |
| NIN | ns | ns | ns | ns |
| NKX2-4 | nd | 4.6 x 10^-7^ (-) | nd | 5.0 x 10^-8^ (-) |
| NPY | ns | ns | ns | ns |
| PIK3CD | 3.9 x 10^-25^ (+) | 6.5 x 10^-29^ (+) | 1.3 x 10^-18^ (+) | 1.9 x 10^-19^ (+) |
| PIM1 | 6.3 x 10^-4^ (-) | 6.2 x 10^-4^ (-) | 3.8 x 10^-2^ (-) | 7.8 x 10^-4^ (-) |
| PRRX2 | 1.3 x 10^-17^ (-) | 3.8 x 10^-13^ (-) | 5.8 x 10^-5^ (-) | 6.2 x 10^-7^ (-) |
| RRAS | 5.8 x 10^-8^ (+) | 1.3 x 10^-9^ (+) | 5.2 x 10^-7^ (+) | 7.9 x 10^-6^ (+) |
| SAGE1 | n | 2.2 x 10^-6^ (-) | n | 5.4 x 10^-6^ (-) |
| SEMA4A | ns | 1.4 x 10^-8^ (+) | ns | 4.4 x 10^-5^ (+) |
| SLC7A3 | ns | ns | ns | ns |
| SSBP3 | 4.9 x 10^-2^ (+) | 6.7 x 10^-12^ (+) | ns | 1.1 x 10^-7^ (+) |
| SURF2 | 4.6 x 10^-2^ (-) | 5.2 x 10^-5^ (-) | ns | 3.8 x 10^-6^ (-) |
| UTF1 | ns | ns | ns | 2.6 x 10^-2^ (-) |
Supplementary Table 3. Significance of high expression of validated genes from the CRISPRa screen in SH-SY5Y cells on overall and event-free survival in Kocak and SEQC NB patient cohorts Numbers indicate Bonferroni-corrected p-values. - = poor survival; + = greater survival; ns = not significant; nd = not in database.

## Slide 13
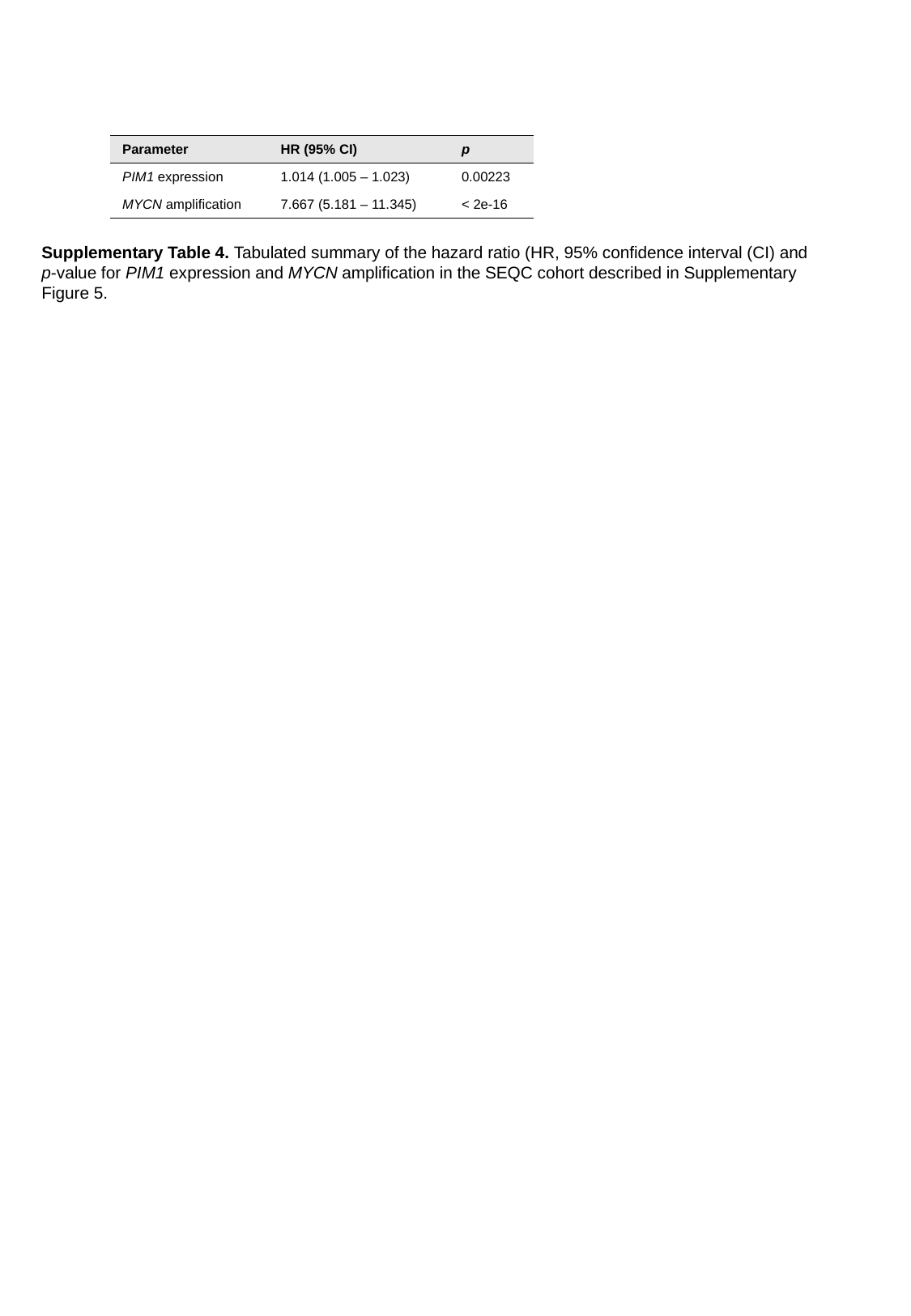

| Parameter | HR (95% CI) | p |
| --- | --- | --- |
| PIM1 expression | 1.014 (1.005 – 1.023) | 0.00223 |
| MYCN amplification | 7.667 (5.181 – 11.345) | < 2e-16 |
Supplementary Table 4. Tabulated summary of the hazard ratio (HR, 95% confidence interval (CI) and p-value for PIM1 expression and MYCN amplification in the SEQC cohort described in Supplementary Figure 5.

## Slide 14
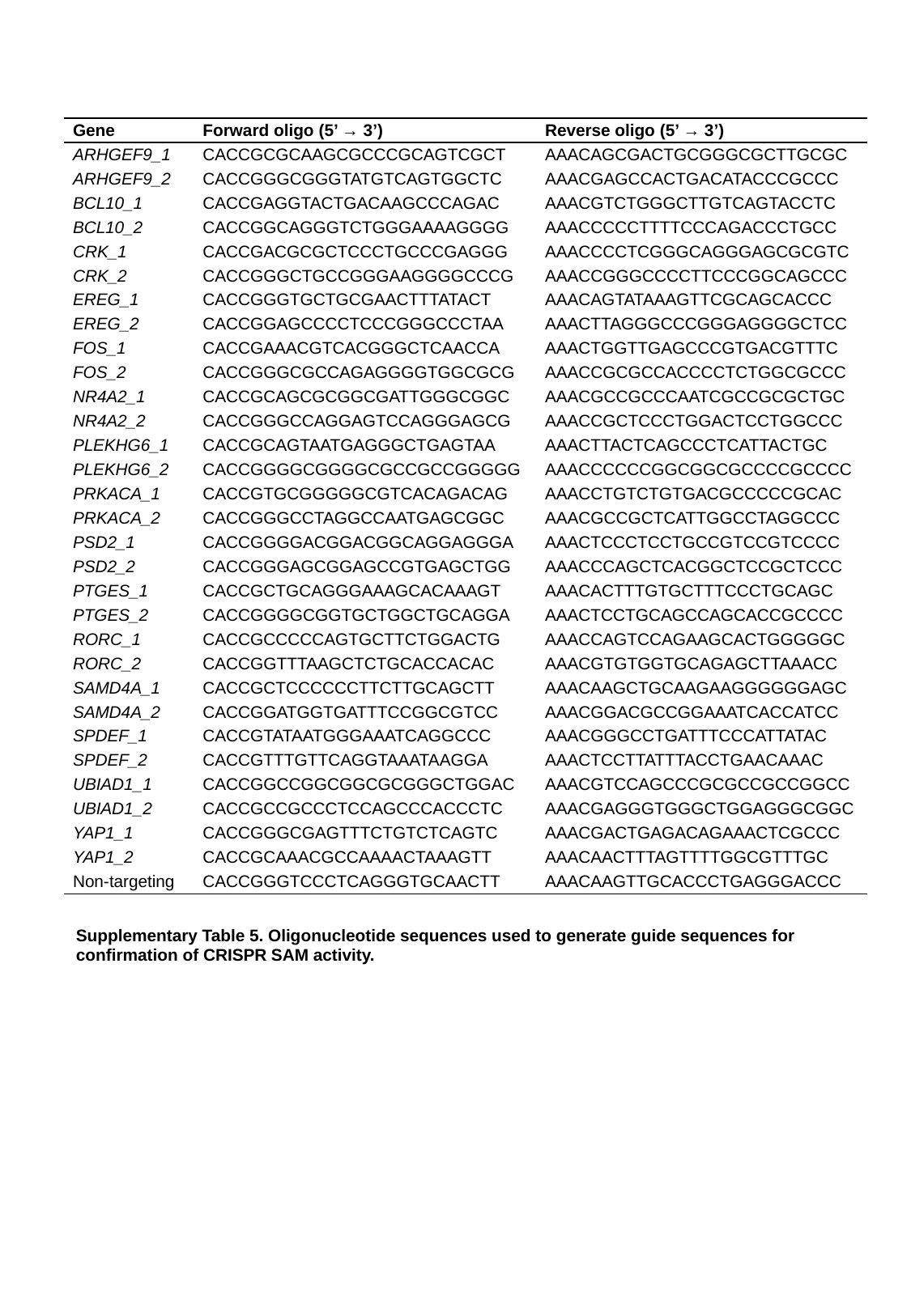

| Gene | Forward oligo (5’ → 3’) | Reverse oligo (5’ → 3’) |
| --- | --- | --- |
| ARHGEF9\_1 | CACCGCGCAAGCGCCCGCAGTCGCT | AAACAGCGACTGCGGGCGCTTGCGC |
| ARHGEF9\_2 | CACCGGGCGGGTATGTCAGTGGCTC | AAACGAGCCACTGACATACCCGCCC |
| BCL10\_1 | CACCGAGGTACTGACAAGCCCAGAC | AAACGTCTGGGCTTGTCAGTACCTC |
| BCL10\_2 | CACCGGCAGGGTCTGGGAAAAGGGG | AAACCCCCTTTTCCCAGACCCTGCC |
| CRK\_1 | CACCGACGCGCTCCCTGCCCGAGGG | AAACCCCTCGGGCAGGGAGCGCGTC |
| CRK\_2 | CACCGGGCTGCCGGGAAGGGGCCCG | AAACCGGGCCCCTTCCCGGCAGCCC |
| EREG\_1 | CACCGGGTGCTGCGAACTTTATACT | AAACAGTATAAAGTTCGCAGCACCC |
| EREG\_2 | CACCGGAGCCCCTCCCGGGCCCTAA | AAACTTAGGGCCCGGGAGGGGCTCC |
| FOS\_1 | CACCGAAACGTCACGGGCTCAACCA | AAACTGGTTGAGCCCGTGACGTTTC |
| FOS\_2 | CACCGGGCGCCAGAGGGGTGGCGCG | AAACCGCGCCACCCCTCTGGCGCCC |
| NR4A2\_1 | CACCGCAGCGCGGCGATTGGGCGGC | AAACGCCGCCCAATCGCCGCGCTGC |
| NR4A2\_2 | CACCGGGCCAGGAGTCCAGGGAGCG | AAACCGCTCCCTGGACTCCTGGCCC |
| PLEKHG6\_1 | CACCGCAGTAATGAGGGCTGAGTAA | AAACTTACTCAGCCCTCATTACTGC |
| PLEKHG6\_2 | CACCGGGGCGGGGCGCCGCCGGGGG | AAACCCCCCGGCGGCGCCCCGCCCC |
| PRKACA\_1 | CACCGTGCGGGGGCGTCACAGACAG | AAACCTGTCTGTGACGCCCCCGCAC |
| PRKACA\_2 | CACCGGGCCTAGGCCAATGAGCGGC | AAACGCCGCTCATTGGCCTAGGCCC |
| PSD2\_1 | CACCGGGGACGGACGGCAGGAGGGA | AAACTCCCTCCTGCCGTCCGTCCCC |
| PSD2\_2 | CACCGGGAGCGGAGCCGTGAGCTGG | AAACCCAGCTCACGGCTCCGCTCCC |
| PTGES\_1 | CACCGCTGCAGGGAAAGCACAAAGT | AAACACTTTGTGCTTTCCCTGCAGC |
| PTGES\_2 | CACCGGGGCGGTGCTGGCTGCAGGA | AAACTCCTGCAGCCAGCACCGCCCC |
| RORC\_1 | CACCGCCCCCAGTGCTTCTGGACTG | AAACCAGTCCAGAAGCACTGGGGGC |
| RORC\_2 | CACCGGTTTAAGCTCTGCACCACAC | AAACGTGTGGTGCAGAGCTTAAACC |
| SAMD4A\_1 | CACCGCTCCCCCCTTCTTGCAGCTT | AAACAAGCTGCAAGAAGGGGGGAGC |
| SAMD4A\_2 | CACCGGATGGTGATTTCCGGCGTCC | AAACGGACGCCGGAAATCACCATCC |
| SPDEF\_1 | CACCGTATAATGGGAAATCAGGCCC | AAACGGGCCTGATTTCCCATTATAC |
| SPDEF\_2 | CACCGTTTGTTCAGGTAAATAAGGA | AAACTCCTTATTTACCTGAACAAAC |
| UBIAD1\_1 | CACCGGCCGGCGGCGCGGGCTGGAC | AAACGTCCAGCCCGCGCCGCCGGCC |
| UBIAD1\_2 | CACCGCCGCCCTCCAGCCCACCCTC | AAACGAGGGTGGGCTGGAGGGCGGC |
| YAP1\_1 | CACCGGGCGAGTTTCTGTCTCAGTC | AAACGACTGAGACAGAAACTCGCCC |
| YAP1\_2 | CACCGCAAACGCCAAAACTAAAGTT | AAACAACTTTAGTTTTGGCGTTTGC |
| Non-targeting | CACCGGGTCCCTCAGGGTGCAACTT | AAACAAGTTGCACCCTGAGGGACCC |
Supplementary Table 5. Oligonucleotide sequences used to generate guide sequences for confirmation of CRISPR SAM activity.

## Slide 15
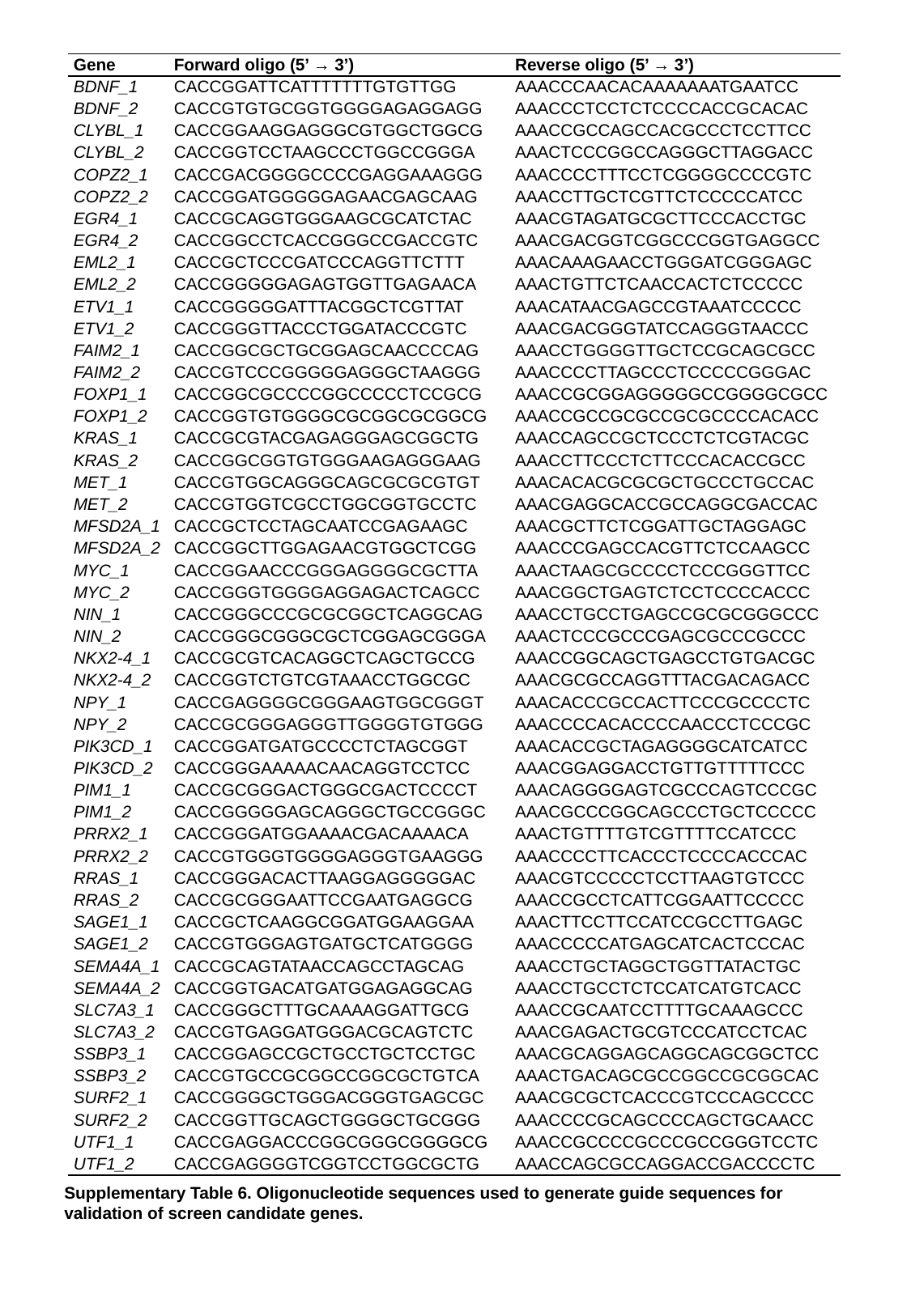

| Gene | Forward oligo (5’ → 3’) | Reverse oligo (5’ → 3’) |
| --- | --- | --- |
| BDNF\_1 | CACCGGATTCATTTTTTTGTGTTGG | AAACCCAACACAAAAAAATGAATCC |
| BDNF\_2 | CACCGTGTGCGGTGGGGAGAGGAGG | AAACCCTCCTCTCCCCACCGCACAC |
| CLYBL\_1 | CACCGGAAGGAGGGCGTGGCTGGCG | AAACCGCCAGCCACGCCCTCCTTCC |
| CLYBL\_2 | CACCGGTCCTAAGCCCTGGCCGGGA | AAACTCCCGGCCAGGGCTTAGGACC |
| COPZ2\_1 | CACCGACGGGGCCCCGAGGAAAGGG | AAACCCCTTTCCTCGGGGCCCCGTC |
| COPZ2\_2 | CACCGGATGGGGGAGAACGAGCAAG | AAACCTTGCTCGTTCTCCCCCATCC |
| EGR4\_1 | CACCGCAGGTGGGAAGCGCATCTAC | AAACGTAGATGCGCTTCCCACCTGC |
| EGR4\_2 | CACCGGCCTCACCGGGCCGACCGTC | AAACGACGGTCGGCCCGGTGAGGCC |
| EML2\_1 | CACCGCTCCCGATCCCAGGTTCTTT | AAACAAAGAACCTGGGATCGGGAGC |
| EML2\_2 | CACCGGGGGAGAGTGGTTGAGAACA | AAACTGTTCTCAACCACTCTCCCCC |
| ETV1\_1 | CACCGGGGGATTTACGGCTCGTTAT | AAACATAACGAGCCGTAAATCCCCC |
| ETV1\_2 | CACCGGGTTACCCTGGATACCCGTC | AAACGACGGGTATCCAGGGTAACCC |
| FAIM2\_1 | CACCGGCGCTGCGGAGCAACCCCAG | AAACCTGGGGTTGCTCCGCAGCGCC |
| FAIM2\_2 | CACCGTCCCGGGGGAGGGCTAAGGG | AAACCCCTTAGCCCTCCCCCGGGAC |
| FOXP1\_1 | CACCGGCGCCCCGGCCCCCTCCGCG | AAACCGCGGAGGGGGCCGGGGCGCC |
| FOXP1\_2 | CACCGGTGTGGGGCGCGGCGCGGCG | AAACCGCCGCGCCGCGCCCCACACC |
| KRAS\_1 | CACCGCGTACGAGAGGGAGCGGCTG | AAACCAGCCGCTCCCTCTCGTACGC |
| KRAS\_2 | CACCGGCGGTGTGGGAAGAGGGAAG | AAACCTTCCCTCTTCCCACACCGCC |
| MET\_1 | CACCGTGGCAGGGCAGCGCGCGTGT | AAACACACGCGCGCTGCCCTGCCAC |
| MET\_2 | CACCGTGGTCGCCTGGCGGTGCCTC | AAACGAGGCACCGCCAGGCGACCAC |
| MFSD2A\_1 | CACCGCTCCTAGCAATCCGAGAAGC | AAACGCTTCTCGGATTGCTAGGAGC |
| MFSD2A\_2 | CACCGGCTTGGAGAACGTGGCTCGG | AAACCCGAGCCACGTTCTCCAAGCC |
| MYC\_1 | CACCGGAACCCGGGAGGGGCGCTTA | AAACTAAGCGCCCCTCCCGGGTTCC |
| MYC\_2 | CACCGGGTGGGGAGGAGACTCAGCC | AAACGGCTGAGTCTCCTCCCCACCC |
| NIN\_1 | CACCGGGCCCGCGCGGCTCAGGCAG | AAACCTGCCTGAGCCGCGCGGGCCC |
| NIN\_2 | CACCGGGCGGGCGCTCGGAGCGGGA | AAACTCCCGCCCGAGCGCCCGCCC |
| NKX2-4\_1 | CACCGCGTCACAGGCTCAGCTGCCG | AAACCGGCAGCTGAGCCTGTGACGC |
| NKX2-4\_2 | CACCGGTCTGTCGTAAACCTGGCGC | AAACGCGCCAGGTTTACGACAGACC |
| NPY\_1 | CACCGAGGGGCGGGAAGTGGCGGGT | AAACACCCGCCACTTCCCGCCCCTC |
| NPY\_2 | CACCGCGGGAGGGTTGGGGTGTGGG | AAACCCCACACCCCAACCCTCCCGC |
| PIK3CD\_1 | CACCGGATGATGCCCCTCTAGCGGT | AAACACCGCTAGAGGGGCATCATCC |
| PIK3CD\_2 | CACCGGGAAAAACAACAGGTCCTCC | AAACGGAGGACCTGTTGTTTTTCCC |
| PIM1\_1 | CACCGCGGGACTGGGCGACTCCCCT | AAACAGGGGAGTCGCCCAGTCCCGC |
| PIM1\_2 | CACCGGGGGAGCAGGGCTGCCGGGC | AAACGCCCGGCAGCCCTGCTCCCCC |
| PRRX2\_1 | CACCGGGATGGAAAACGACAAAACA | AAACTGTTTTGTCGTTTTCCATCCC |
| PRRX2\_2 | CACCGTGGGTGGGGAGGGTGAAGGG | AAACCCCTTCACCCTCCCCACCCAC |
| RRAS\_1 | CACCGGGACACTTAAGGAGGGGGAC | AAACGTCCCCCTCCTTAAGTGTCCC |
| RRAS\_2 | CACCGCGGGAATTCCGAATGAGGCG | AAACCGCCTCATTCGGAATTCCCCC |
| SAGE1\_1 | CACCGCTCAAGGCGGATGGAAGGAA | AAACTTCCTTCCATCCGCCTTGAGC |
| SAGE1\_2 | CACCGTGGGAGTGATGCTCATGGGG | AAACCCCCATGAGCATCACTCCCAC |
| SEMA4A\_1 | CACCGCAGTATAACCAGCCTAGCAG | AAACCTGCTAGGCTGGTTATACTGC |
| SEMA4A\_2 | CACCGGTGACATGATGGAGAGGCAG | AAACCTGCCTCTCCATCATGTCACC |
| SLC7A3\_1 | CACCGGGCTTTGCAAAAGGATTGCG | AAACCGCAATCCTTTTGCAAAGCCC |
| SLC7A3\_2 | CACCGTGAGGATGGGACGCAGTCTC | AAACGAGACTGCGTCCCATCCTCAC |
| SSBP3\_1 | CACCGGAGCCGCTGCCTGCTCCTGC | AAACGCAGGAGCAGGCAGCGGCTCC |
| SSBP3\_2 | CACCGTGCCGCGGCCGGCGCTGTCA | AAACTGACAGCGCCGGCCGCGGCAC |
| SURF2\_1 | CACCGGGGCTGGGACGGGTGAGCGC | AAACGCGCTCACCCGTCCCAGCCCC |
| SURF2\_2 | CACCGGTTGCAGCTGGGGCTGCGGG | AAACCCCGCAGCCCCAGCTGCAACC |
| UTF1\_1 | CACCGAGGACCCGGCGGGCGGGGCG | AAACCGCCCCGCCCGCCGGGTCCTC |
| UTF1\_2 | CACCGAGGGGTCGGTCCTGGCGCTG | AAACCAGCGCCAGGACCGACCCCTC |
Supplementary Table 6. Oligonucleotide sequences used to generate guide sequences for validation of screen candidate genes.

## Slide 16
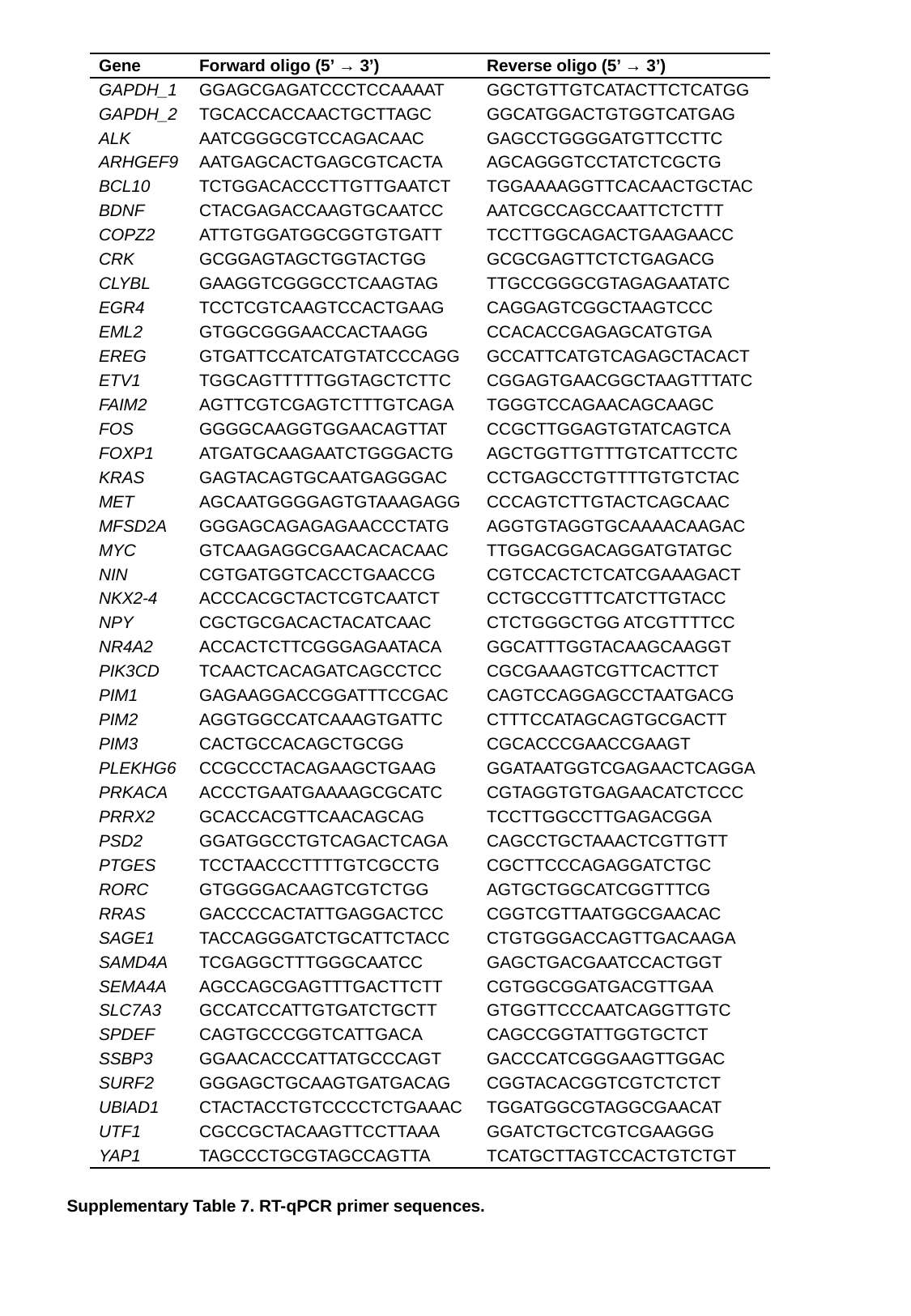

| Gene | Forward oligo (5’ → 3’) | Reverse oligo (5’ → 3’) |
| --- | --- | --- |
| GAPDH\_1 | GGAGCGAGATCCCTCCAAAAT | GGCTGTTGTCATACTTCTCATGG |
| GAPDH\_2 | TGCACCACCAACTGCTTAGC | GGCATGGACTGTGGTCATGAG |
| ALK | AATCGGGCGTCCAGACAAC | GAGCCTGGGGATGTTCCTTC |
| ARHGEF9 | AATGAGCACTGAGCGTCACTA | AGCAGGGTCCTATCTCGCTG |
| BCL10 | TCTGGACACCCTTGTTGAATCT | TGGAAAAGGTTCACAACTGCTAC |
| BDNF | CTACGAGACCAAGTGCAATCC | AATCGCCAGCCAATTCTCTTT |
| COPZ2 | ATTGTGGATGGCGGTGTGATT | TCCTTGGCAGACTGAAGAACC |
| CRK | GCGGAGTAGCTGGTACTGG | GCGCGAGTTCTCTGAGACG |
| CLYBL | GAAGGTCGGGCCTCAAGTAG | TTGCCGGGCGTAGAGAATATC |
| EGR4 | TCCTCGTCAAGTCCACTGAAG | CAGGAGTCGGCTAAGTCCC |
| EML2 | GTGGCGGGAACCACTAAGG | CCACACCGAGAGCATGTGA |
| EREG | GTGATTCCATCATGTATCCCAGG | GCCATTCATGTCAGAGCTACACT |
| ETV1 | TGGCAGTTTTTGGTAGCTCTTC | CGGAGTGAACGGCTAAGTTTATC |
| FAIM2 | AGTTCGTCGAGTCTTTGTCAGA | TGGGTCCAGAACAGCAAGC |
| FOS | GGGGCAAGGTGGAACAGTTAT | CCGCTTGGAGTGTATCAGTCA |
| FOXP1 | ATGATGCAAGAATCTGGGACTG | AGCTGGTTGTTTGTCATTCCTC |
| KRAS | GAGTACAGTGCAATGAGGGAC | CCTGAGCCTGTTTTGTGTCTAC |
| MET | AGCAATGGGGAGTGTAAAGAGG | CCCAGTCTTGTACTCAGCAAC |
| MFSD2A | GGGAGCAGAGAGAACCCTATG | AGGTGTAGGTGCAAAACAAGAC |
| MYC | GTCAAGAGGCGAACACACAAC | TTGGACGGACAGGATGTATGC |
| NIN | CGTGATGGTCACCTGAACCG | CGTCCACTCTCATCGAAAGACT |
| NKX2-4 | ACCCACGCTACTCGTCAATCT | CCTGCCGTTTCATCTTGTACC |
| NPY | CGCTGCGACACTACATCAAC | CTCTGGGCTGG ATCGTTTTCC |
| NR4A2 | ACCACTCTTCGGGAGAATACA | GGCATTTGGTACAAGCAAGGT |
| PIK3CD | TCAACTCACAGATCAGCCTCC | CGCGAAAGTCGTTCACTTCT |
| PIM1 | GAGAAGGACCGGATTTCCGAC | CAGTCCAGGAGCCTAATGACG |
| PIM2 | AGGTGGCCATCAAAGTGATTC | CTTTCCATAGCAGTGCGACTT |
| PIM3 | CACTGCCACAGCTGCGG | CGCACCCGAACCGAAGT |
| PLEKHG6 | CCGCCCTACAGAAGCTGAAG | GGATAATGGTCGAGAACTCAGGA |
| PRKACA | ACCCTGAATGAAAAGCGCATC | CGTAGGTGTGAGAACATCTCCC |
| PRRX2 | GCACCACGTTCAACAGCAG | TCCTTGGCCTTGAGACGGA |
| PSD2 | GGATGGCCTGTCAGACTCAGA | CAGCCTGCTAAACTCGTTGTT |
| PTGES | TCCTAACCCTTTTGTCGCCTG | CGCTTCCCAGAGGATCTGC |
| RORC | GTGGGGACAAGTCGTCTGG | AGTGCTGGCATCGGTTTCG |
| RRAS | GACCCCACTATTGAGGACTCC | CGGTCGTTAATGGCGAACAC |
| SAGE1 | TACCAGGGATCTGCATTCTACC | CTGTGGGACCAGTTGACAAGA |
| SAMD4A | TCGAGGCTTTGGGCAATCC | GAGCTGACGAATCCACTGGT |
| SEMA4A | AGCCAGCGAGTTTGACTTCTT | CGTGGCGGATGACGTTGAA |
| SLC7A3 | GCCATCCATTGTGATCTGCTT | GTGGTTCCCAATCAGGTTGTC |
| SPDEF | CAGTGCCCGGTCATTGACA | CAGCCGGTATTGGTGCTCT |
| SSBP3 | GGAACACCCATTATGCCCAGT | GACCCATCGGGAAGTTGGAC |
| SURF2 | GGGAGCTGCAAGTGATGACAG | CGGTACACGGTCGTCTCTCT |
| UBIAD1 | CTACTACCTGTCCCCTCTGAAAC | TGGATGGCGTAGGCGAACAT |
| UTF1 | CGCCGCTACAAGTTCCTTAAA | GGATCTGCTCGTCGAAGGG |
| YAP1 | TAGCCCTGCGTAGCCAGTTA | TCATGCTTAGTCCACTGTCTGT |
Supplementary Table 7. RT-qPCR primer sequences.

## Slide 17
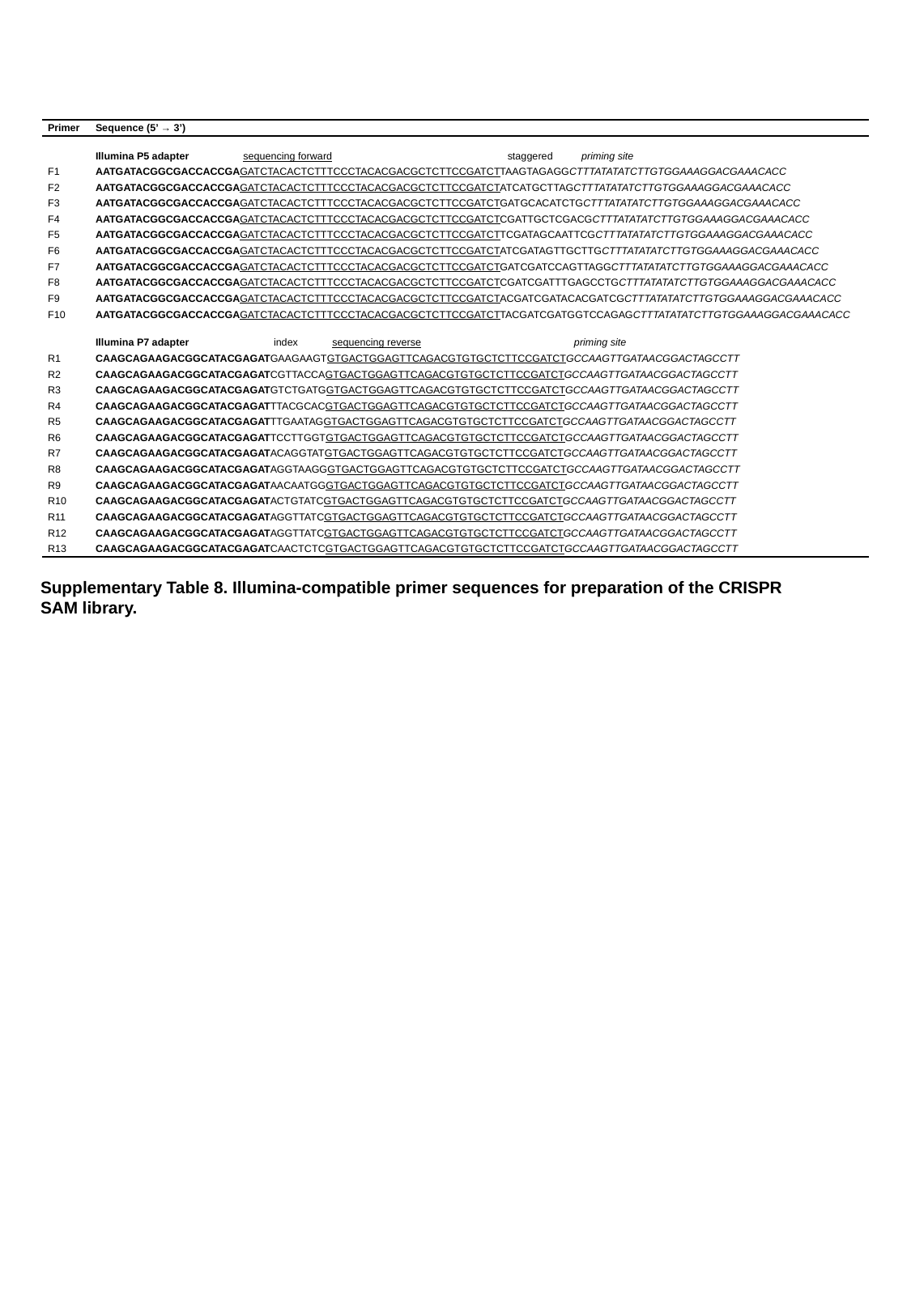

| Primer | Sequence (5’ → 3’) |
| --- | --- |
| | |
| | Illumina P5 adapter sequencing forward staggered priming site |
| F1 | AATGATACGGCGACCACCGAGATCTACACTCTTTCCCTACACGACGCTCTTCCGATCTTAAGTAGAGGCTTTATATATCTTGTGGAAAGGACGAAACACC |
| F2 | AATGATACGGCGACCACCGAGATCTACACTCTTTCCCTACACGACGCTCTTCCGATCTATCATGCTTAGCTTTATATATCTTGTGGAAAGGACGAAACACC |
| F3 | AATGATACGGCGACCACCGAGATCTACACTCTTTCCCTACACGACGCTCTTCCGATCTGATGCACATCTGCTTTATATATCTTGTGGAAAGGACGAAACACC |
| F4 | AATGATACGGCGACCACCGAGATCTACACTCTTTCCCTACACGACGCTCTTCCGATCTCGATTGCTCGACGCTTTATATATCTTGTGGAAAGGACGAAACACC |
| F5 | AATGATACGGCGACCACCGAGATCTACACTCTTTCCCTACACGACGCTCTTCCGATCTTCGATAGCAATTCGCTTTATATATCTTGTGGAAAGGACGAAACACC |
| F6 | AATGATACGGCGACCACCGAGATCTACACTCTTTCCCTACACGACGCTCTTCCGATCTATCGATAGTTGCTTGCTTTATATATCTTGTGGAAAGGACGAAACACC |
| F7 | AATGATACGGCGACCACCGAGATCTACACTCTTTCCCTACACGACGCTCTTCCGATCTGATCGATCCAGTTAGGCTTTATATATCTTGTGGAAAGGACGAAACACC |
| F8 | AATGATACGGCGACCACCGAGATCTACACTCTTTCCCTACACGACGCTCTTCCGATCTCGATCGATTTGAGCCTGCTTTATATATCTTGTGGAAAGGACGAAACACC |
| F9 | AATGATACGGCGACCACCGAGATCTACACTCTTTCCCTACACGACGCTCTTCCGATCTACGATCGATACACGATCGCTTTATATATCTTGTGGAAAGGACGAAACACC |
| F10 | AATGATACGGCGACCACCGAGATCTACACTCTTTCCCTACACGACGCTCTTCCGATCTTACGATCGATGGTCCAGAGCTTTATATATCTTGTGGAAAGGACGAAACACC |
| | |
| | Illumina P7 adapter index sequencing reverse priming site |
| R1 | CAAGCAGAAGACGGCATACGAGATGAAGAAGTGTGACTGGAGTTCAGACGTGTGCTCTTCCGATCTGCCAAGTTGATAACGGACTAGCCTT |
| R2 | CAAGCAGAAGACGGCATACGAGATCGTTACCAGTGACTGGAGTTCAGACGTGTGCTCTTCCGATCTGCCAAGTTGATAACGGACTAGCCTT |
| R3 | CAAGCAGAAGACGGCATACGAGATGTCTGATGGTGACTGGAGTTCAGACGTGTGCTCTTCCGATCTGCCAAGTTGATAACGGACTAGCCTT |
| R4 | CAAGCAGAAGACGGCATACGAGATTTACGCACGTGACTGGAGTTCAGACGTGTGCTCTTCCGATCTGCCAAGTTGATAACGGACTAGCCTT |
| R5 | CAAGCAGAAGACGGCATACGAGATTTGAATAGGTGACTGGAGTTCAGACGTGTGCTCTTCCGATCTGCCAAGTTGATAACGGACTAGCCTT |
| R6 | CAAGCAGAAGACGGCATACGAGATTCCTTGGTGTGACTGGAGTTCAGACGTGTGCTCTTCCGATCTGCCAAGTTGATAACGGACTAGCCTT |
| R7 | CAAGCAGAAGACGGCATACGAGATACAGGTATGTGACTGGAGTTCAGACGTGTGCTCTTCCGATCTGCCAAGTTGATAACGGACTAGCCTT |
| R8 | CAAGCAGAAGACGGCATACGAGATAGGTAAGGGTGACTGGAGTTCAGACGTGTGCTCTTCCGATCTGCCAAGTTGATAACGGACTAGCCTT |
| R9 | CAAGCAGAAGACGGCATACGAGATAACAATGGGTGACTGGAGTTCAGACGTGTGCTCTTCCGATCTGCCAAGTTGATAACGGACTAGCCTT |
| R10 | CAAGCAGAAGACGGCATACGAGATACTGTATCGTGACTGGAGTTCAGACGTGTGCTCTTCCGATCTGCCAAGTTGATAACGGACTAGCCTT |
| R11 | CAAGCAGAAGACGGCATACGAGATAGGTTATCGTGACTGGAGTTCAGACGTGTGCTCTTCCGATCTGCCAAGTTGATAACGGACTAGCCTT |
| R12 | CAAGCAGAAGACGGCATACGAGATAGGTTATCGTGACTGGAGTTCAGACGTGTGCTCTTCCGATCTGCCAAGTTGATAACGGACTAGCCTT |
| R13 | CAAGCAGAAGACGGCATACGAGATCAACTCTCGTGACTGGAGTTCAGACGTGTGCTCTTCCGATCTGCCAAGTTGATAACGGACTAGCCTT |
Supplementary Table 8. Illumina-compatible primer sequences for preparation of the CRISPR SAM library.
