# Supplementary material for: The targetable kinase PIM1 drives ALK inhibitor resistance in high-risk neuroblastoma independent of MYCN status
Source: Nat Commun. 2019 Nov 28;10:5428. doi: 10.1038/s41467-019-13315-x (PMC6883072; doi:10.1038/s41467-019-13315-x)
Supplement: Supplementary file 2 — Description of Additional Supplementary Files [file 41467_2019_13315_MOESM2_ESM.docx]

Description of Additional Supplementary Files

**Supplementary Data 1:** Raw gRNA read counts for the CRISPRa screen performed in SH-SY5Y cells

**Supplementary Data 2:** Raw gRNA read counts for the CRISPRa screen performed in CHLA-20 cells
