# Supplementary material for: The targetable kinase PIM1 drives ALK inhibitor resistance in high-risk neuroblastoma independent of MYCN status
Source: Nat Commun. 2019 Nov 28;10:5428. doi: 10.1038/s41467-019-13315-x (PMC6883072; doi:10.1038/s41467-019-13315-x)
Supplement: Supplementary file 6 — Reporting Summary [file 41467_2019_13315_MOESM6_ESM.pdf]

nature research

Corresponding author(s): Suzanne Turner

Last updated by author(s): Oct 9, 2019

Reporting Summary

Nature Research wishes to improve the reproducibility of the work that we publish. This form provides structure for consistency and transparency in reporting. For further information on Nature Research policies, see Authors & Referees and the Editorial Policy Checklist.

Statistics

For all statistical analyses, confirm that the following items are present in the figure legend, table legend, main text, or Methods section.

n/a	Confirmed	
☐	☒	The exact sample size (*n*) for each experimental group/condition, given as a discrete number and unit of measurement
☐	☒	A statement on whether measurements were taken from distinct samples or whether the same sample was measured repeatedly
☐	☒	The statistical test(s) used AND whether they are one- or two-sided<br>*Only common tests should be described solely by name; describe more complex techniques in the Methods section.*
☒	☐	A description of all covariates tested
☒	☐	A description of any assumptions or corrections, such as tests of normality and adjustment for multiple comparisons
☐	☒	A full description of the statistical parameters including central tendency (e.g. means) or other basic estimates (e.g. regression coefficient) AND variation (e.g. standard deviation) or associated estimates of uncertainty (e.g. confidence intervals)
☐	☒	For null hypothesis testing, the test statistic (e.g. *F*, *t*, *r*) with confidence intervals, effect sizes, degrees of freedom and *P* value noted<br>*Give P values as exact values whenever suitable.*
☒	☐	For Bayesian analysis, information on the choice of priors and Markov chain Monte Carlo settings
☒	☐	For hierarchical and complex designs, identification of the appropriate level for tests and full reporting of outcomes
☐	☒	Estimates of effect sizes (e.g. Cohen's *d*, Pearson's *r*), indicating how they were calculated

Our web collection on statistics for biologists contains articles on many of the points above.

Software and code

Policy information about availability of computer code

Data collection	Microplate data were collected using SoftMax Pro v7 software. RT-qPCR data were collected using QuantStudio v1.1 software.
Data analysis	GraphPad Prism v7 software. FastQC was used to evaluate quality of sequencing reads for CRISPR SAM screens. GSEA Desktop v3.0 software was used to conduct gene set enrichment analysis on hit genes from SH-SY5Y CRISPR SAM screen. R2: Genomics Analysis and Visualization Platform (http://r2.amc.nl) was used to conduct Kaplan-Meier survival analysis in public neuroblastoma patient datasets.

For manuscripts utilizing custom algorithms or software that are central to the research but not yet described in published literature, software must be made available to editors/reviewers. We strongly encourage code deposition in a community repository (e.g. GitHub). See the Nature Research guidelines for submitting code & software for further information.

Data

Policy information about availability of data

All manuscripts must include a data availability statement. This statement should provide the following information, where applicable:

- Accession codes, unique identifiers, or web links for publicly available datasets
- A list of figures that have associated raw data
- A description of any restrictions on data availability

The authors declare that the data supporting the findings of this study are available within the paper and its supplementary information files.

Field-specific reporting

Please select the one below that is the best fit for your research. If you are not sure, read the appropriate sections before making your selection.

☒ Life sciences ☐ Behavioural & social sciences ☐ Ecological, evolutionary & environmental sciences

For a reference copy of the document with all sections, see nature.com/documents/nr-reporting-summary-flat.pdf

Life sciences study design

All studies must disclose on these points even when the disclosure is negative.

Sample size	For mouse PDX studies, we used a power calculation and determined that a group size of 4 mice was sufficient to generate statistical significance between groups while minimising animal use.
Data exclusions	No data were excluded from this publication.
Replication	All experiments were replicated at least twice. We can confirm that all data presented in this publication were reproducible.
Randomization	Mice were allocated into treatment groups in a manner that was blind to sex and baseline weight.
Blinding	The individuals dosing the mice and recording weight and tumour size did not have knowledge of the predicted result.

Reporting for specific materials, systems and methods

We require information from authors about some types of materials, experimental systems and methods used in many studies. Here, indicate whether each material, system or method listed is relevant to your study. If you are not sure if a list item applies to your research, read the appropriate section before selecting a response.

Materials & experimental systems

n/a	Involved in the study	
☐	☒	Antibodies
☐	☒	Eukaryotic cell lines
☒	☐	Palaeontology
☐	☒	Animals and other organisms
☒	☐	Human research participants
☒	☐	Clinical data

Methods

n/a	Involved in the study	
☒	☐	ChIP-seq
☐	☒	Flow cytometry
☒	☐	MRI-based neuroimaging

Antibodies

Antibodies used	The following antibodies were all purchased from Cell Signaling Technology (CST): anti-BAD (Cat#9292, Lot#13), anti-phospho-BAD (Ser112) (Cat#9291, Lot#8), anti-BAX (Cat#5023, Lot#2), anti-BCL-2 (Cat#4223, Lot#4), anti-PIM1 (Cat#2907, Lot#3), anti-PRAS40 (Cat#2691, Lot#8), anti-phospho-PRAS40 (Thr246) (Cat#13175, Lot#1), and anti-α-tubulin (Cat#T9026, Lot#22).
Validation	Taken from the Cell Signaling Technology website:<br><br>"Every CST antibody undergoes rigorous application-specific validation testing customized according to the target and needs of the individual antibody. Below is a sampling of the methods we may employ to ensure our antibodies perform as expected.<br><br>1. Materials: Verifying Specificity, Sensitivity, and Reproducibility<br>- Analysis of a large panel of cell lines with known target expression levels<br>- Treatment of cells with appropriate kinase-specific activators and/or inhibitors<br>- Phosphatase treatment<br>- Correct subcellular localization or treatment-induced translocation<br>- Comparison of results with antibody and isotype control to ensure acceptable signal-to-background ratio<br>- Target-specific signal verified in transfected cells, knockout cells, or siRNA-treated cells<br>- Blocking with antigen peptide to confirm elimination of specific signal<br>- Side-by-side comparison of a new lot with previous lots to ensure lot-to-lot consistency<br>2. Methods: Identifying Optimal Conditions<br>- Optimal dilutions and buffers predetermined<br>- Positive and negative control cell extracts specified<br>- Detailed protocols already optimized<br>- Treatment of cells with appropriate kinase-specific activators and/or inhibitors<br>- Phosphatase treatment<br>- Correct subcellular localization or treatment-induced translocation

- Comparison of results with antibody and isotype control to ensure acceptable signal-to-background ratio
- Target-specific signal verified in transfected cells, knockout cells, or siRNA-treated cells
- Blocking with antigen peptide to confirm elimination of specific signal
- Side-by-side comparison of a new lot with previous lots to ensure lot-to-lot consistency"

Eukaryotic cell lines

Policy information about cell lines

Cell line source(s)	CHLA-15, CHLA-20, CHLA-42, CHLA-90, CHLA-95, CHLA-171, COG-N-426 (Felix), LA-N-5, LA-N-6, NB-1643, NB-EBC1, SK-N-BE(1), SK-N-BE(2), SK-N-FI, SMS-KAN, SMS-KCR and SMS-LHN were obtained from Childhood Cancer Repository (www.cccells.org). CHP-134, IMR-32, KELLY, LA-N-1 and SH-SY5Y were obtained from the European Collection of Authenticated Cell Cultures (ECACC). GI-ME-N, NBL-S and NGP were obtained from German Collection of Microorganisms and Cell Cultures (DSMZ) and 293FT was obtained from Thermo Fisher Scientific.
Authentication	All cell lines were authenticated by STR profiling at the repositories.
Mycoplasma contamination	All cell lines were tested quarterly for Mycoplasma contamination during the project and were negative.
Commonly misidentified lines (See ICLAC register)	None

Animals and other organisms

Policy information about studies involving animals; ARRIVE guidelines recommended for reporting animal research

Laboratory animals	Nod SCID Gamma mice (Charles River) were used. Ages ranged from 6-8 weeks at the start of the study and were a random mix of males and females.
Wild animals	N/A
Field-collected samples	N/A
Ethics oversight	Ethical approval was sought and obtained from the University of Cambridge Animal Welfare and Ethical Review Board (AWERB). All experiments were conducted in accordance with the Animals (Scientific Procedures) Act 1986 under the Project Licence P4DBEFF63.

Note that full information on the approval of the study protocol must also be provided in the manuscript.

Flow Cytometry

Plots

Confirm that:

☒ The axis labels state the marker and fluorochrome used (e.g. CD4-FITC).

☒ The axis scales are clearly visible. Include numbers along axes only for bottom left plot of group (a 'group' is an analysis of identical markers).

☒ All plots are contour plots with outliers or pseudocolor plots.

☒ A numerical value for number of cells or percentage (with statistics) is provided.

Methodology

Sample preparation	Cells were centrifuged, washed once in PBS, then resuspended in 100 µL of Annexin V Binding Buffer containing 5 µL/reaction APC-Annexin V (BioLegend, Cat#640920) and incubated for 30 minutes at room temperature. Afterwards, APC-Annexin V was removed from the cells by centrifugation, washed once in Annexin V Binding Buffer (Thermo Fisher Scientific, Cat#V13246), then cells resuspended in 100 µL of Annexin V Binding Buffer containing 1 mg/mL propidium iodide (Sigma-Aldrich, Cat#P4170-10MG).
Instrument	BD Accuri C6 flow cytometer
Software	FlowJo V10 software
Cell population abundance	N/A
Gating strategy	Cells were gated according to physical parameters in order to discard cell debris (FCS/SSC) and cell clumps (Width/Area). Healthy cells were excluded by selecting the PI- and Annexin V-positive cell population.

☒ Tick this box to confirm that a figure exemplifying the gating strategy is provided in the Supplementary Information.
